# Past environmental changes affected lemur population dynamics prior to human impact in Madagascar

**DOI:** 10.1038/s42003-021-02620-1

**Published:** 2021-09-15

**Authors:** Helena Teixeira, Vincent Montade, Jordi Salmona, Julia Metzger, Laurent Bremond, Thomas Kasper, Gerhard Daut, Sylvie Rouland, Sandratrinirainy Ranarilalatiana, Romule Rakotondravony, Lounès Chikhi, Hermann Behling, Ute Radespiel

**Affiliations:** 1 Institute of Zoology, University of Veterinary Medicine Hannover, Bünteweg 17, 30559 Hannover, Germany; 2 University of Goettingen, Department of Palynology and Climate Dynamics, Untere Karspüle 2, 37073 Goettingen, Germany; 3 ISEM, Université Montpellier, CNRS, IRD, EPHE, Place Eugène Bataillon, Montpellier, France; 4 CNRS-UPS-IRD, UMR5174, Laboratoire Évolution & Diversité Biologique, Université Paul Sabatier, 118 route de Narbonne, 31062 Toulouse, France; 5 Institute of Animal Breeding and Genetics, University of Veterinary Medicine Hannover, Bünteweg 17p, 30559 Hannover, Germany; 6 Veterinary Functional Genomics, Max Planck Institute for Molecular Genetics, Ihnestrasse 73, 14195 Berlin, Germany; 7 Friedrich-Schiller-University Jena, Department of Physical Geography, Loebdergraben 32, 07743 Jena, Germany; 8 Université d’Antananarivo, Faculté des Sciences, Mention Biologie et Ecologie Végétale, Laboratoire de Palynologie Appliquée, B.P 905 - 101, Antananarivo, Madagascar; 9 Ecole Doctorale Ecosystèmes Naturels (EDEN), University of Mahajanga, 5 Rue Georges V - Immeuble KAKAL, Mahajanga Be, B.P. 652, Mahajanga, 401 Madagascar; 10 Faculté des Sciences, de Technologies et de l’Environnement, University of Mahajanga, 5 Rue Georges V - Immeuble KAKAL, Mahajanga Be, B.P. 652, Mahajanga, 401 Madagascar; 11 Instituto Gulbenkian de Ciência, Rua da Quinta Grande, 6, P-2780-156 Oeiras, Portugal; 12 Laboratoire Évolution & Diversité Biologique (EDB UMR 5174), Université de Toulouse Midi-Pyrénées, CNRS, IRD, UPS, 118 route de Narbonne, Bât. 4R1, 31062 Toulouse cedex 9, France

**Keywords:** Population genetics, Palaeoecology

## Abstract

Quaternary climatic changes have been invoked as important drivers of species diversification worldwide. However, the impact of such changes on vegetation and animal population dynamics in tropical regions remains debated. To overcome this uncertainty, we integrated high-resolution paleoenvironmental reconstructions from a sedimentary record covering the past 25,000 years with demographic inferences of a forest-dwelling primate species (*Microcebus arnholdi*), in northern Madagascar. Result comparisons suggest that climate changes through the African Humid Period (15.2 – 5.5 kyr) strongly affected the demographic dynamics of *M. arnholdi*. We further inferred a population decline in the last millennium which was likely shaped by the combination of climatic and anthropogenic impacts. Our findings demonstrate that population fluctuations in Malagasy wildlife were substantial prior to a significant human impact. This provides a critical knowledge of climatically driven, environmental and ecological changes in the past, which is essential to better understand the dynamics and resilience of current biodiversity.

## Abstract (web summary)

Teixeira et al. combine genomic and palaeoecological datasets to infer the demographic history of a forest-dwelling lemur in Madagascar. Their findings indicate that historical environmental changes drove population demographic changes prior to anthropogenic impact, which was detected in the study region during the last millennium.

## Introduction

Past climate changes have often been invoked as important drivers of population demographic dynamics, species distribution, and genomic diversity patterns, as species directly respond to environmental changes and to changes in resource availability^1^. Mainly studied in temperate regions^2–5^, such dynamics remain much less explored and more debated in the tropics^6,7^, which are characterized by a very high biodiversity richness. Among tropical regions, Madagascar, with its long history of isolation, high rates of endemism^8,9^, and no significant human impact until the late Holocene^10^, is a key model area to understand how natural climate changes impacted evolutionary trajectories. Past climate fluctuations have been proposed as one of the main drivers of species diversification on the island, for example in lemurs, amphibians, or reptiles^11–14^. Mouse lemurs (*Microcebus* spp.) are the world’s smallest extant primates and form a speciose genus among lemurs with 25 currently recognized species^15^. They represent a suitable model system to investigate the impact of past environmental changes on demographic dynamics because (i) they are forest-dependent and should therefore be strongly influenced by vegetation changes; (ii) they have a comparably short generation time and a high reproductive rate, thereby quickly accumulating genetic signatures of past demographic events; and (iii) populations are still large enough and may have preserved enough genetic diversity to accurately reconstruct their demographic history^11,16,17^. A wide range of molecular approaches have been recently developed to infer species demographic history using genomic data^18–23^. However, interpretations are challenging, given the overall lack of historic population data and the scarcity of paleoenvironmental reconstructions. In particular, the evaluation of realistic demographic scenarios (e.g., combining population size and population structure) requires information about past habitat fluctuations that can only be provided by local paleoenvironmental reconstructions.

**Fig. 1:**
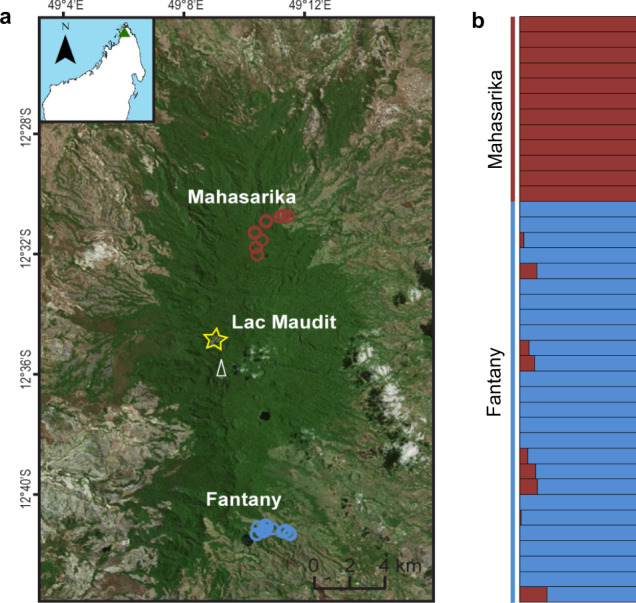
Study area, sampling strategy, and population genetic structure. **a** Location of the study area in Montagne d’Ambre, in northern Madagascar showing the northern (1073 m asl) and southern site (848 m asl) for mouse lemur sampling (red and blue circles) and the study site for lacustrine sediment coring at 1250 m asl (star). All three sites were within 18 km of each other. The white triangle represents the mountaintop. The distribution area was drawn based on resources available in the public domain *Microsoft Bing Virtual Earth*. Individual coordinates can be found in Supplementary Table S2. **b** Assignment of 38 individuals to two genetic clusters (*K* = 2) using genotype likelihoods. Each individual is represented by a single horizontal bar and each color represents a distinct genetic cluster. Samples are sorted by sampling site. The animals sampled in the northern and southern site were assigned to two distinct genetic clusters with low levels of admixture between the two sites.

To investigate how past environmental changes shaped current biodiversity in a tropical rainforest ecosystem, we combined high-resolution paleoenvironmental reconstructions and demographic modeling of mouse lemur genomics data generated for the same study area, Montagne d’Ambre National Park (NP, Fig. 1a). Located in northern Madagascar, this NP fulfills two essential prerequisites: (i) mouse lemurs are known to occur in the evergreen humid forest of this NP^24^ and (ii) this NP hosts volcanic (maar) lakes. Such maar lakes are well known as ideally suited archives as they continuously accumulate sediments, which serve as natural recorders of local and regional environmental and climate dynamics. Since the maar lakes in Montagne d’Ambre are at least of Neogene or Quaternary age, their deposits are assumed to reflect changes in the climatic and environmental conditions during critical periods such as the Pleistocene–Holocene transition. This transition led to abrupt climate and vegetation changes in mainland Africa through the so-called African Humid Period (AHP)^25,26^. Using genome-wide data, four complementary demographic approaches (*Stairway Plot, PSMC, IICR* simulations, and *fastsimcoal2*) were used to infer past population size and connectivity changes of *Microcebus arnholdi* in two parts of the NP, about 18 km from each other. Specifically, mouse lemur dynamics were studied in the northern part of the NP inside the evergreen humid forest (Mahasarika, 1073 m asl, Fig. 1a) and in the southern part of the NP along the forest-grassland ecotone (Fantany, 848 m asl, Fig. 1a). The inferred demographic dynamics were then compared and integrated with paleoenvironmental reconstructions that were derived from a continuous sediment record from Lac Maudit, located at the center of the NP in the evergreen humid forest (1250 m asl Fig. 1a) and less than 6 km away from either mouse lemur site. Such a dual paleoecological–genomic approach that compares genomic inferences with predictions derived from local paleoenvironmental datasets is unique for tropical regions. Our result comparisons demonstrate that the demographic dynamics of *M. arnholdi* were exclusively shaped by natural climate changes until the late Holocene, whereas the dynamics during the last millennium were likely shaped by both natural climate and anthropogenic changes. Altogether, our study illuminates how natural climate fluctuations and anthropogenic activities impacted Malagasy wildlife across time and provides a critical understanding of biological responses of forest-dwelling species to ongoing and accelerating climate change.

## Results and discussion

### Five periods of distinct environmental conditions during the past 25 kyr

**Fig. 2:**
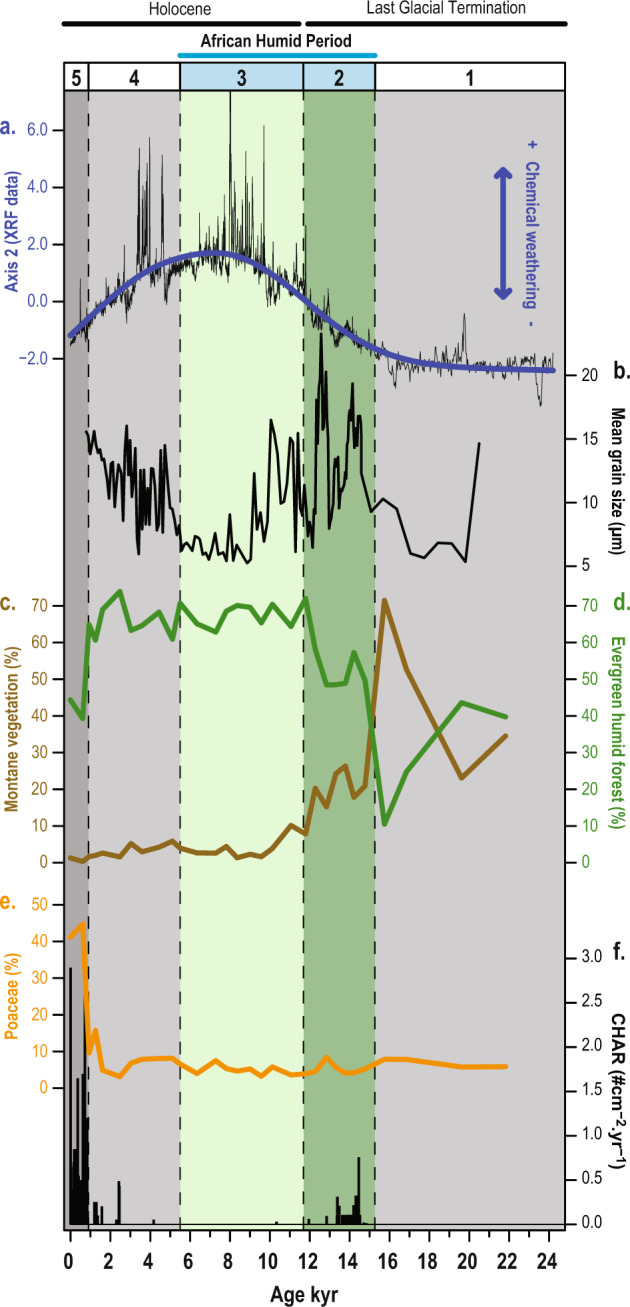
Synthetic paleoenvironmental reconstructions obtained from the sediment core recovered in Lac Maudit. **a** Axis 2 of the Principal Component Analysis performed on the X-ray fluorescence (XRF) scanning record, **b** mean-grain size measurements, **c** pollen sum of specific taxa from montane vegetation (Ericaceae, *Myrica*, *Podocarpus*), **d** pollen sum of specific taxa from evergreen humid forest (Myrtaceae, *Elaeocarpus*, *Macaranga-Mallotus*, *Acalypha*, *Weinmannia*, Moraceae-Urticaceae, *Celtis*, Araliaceae, *Trema*, *Ilex*, *Noronhia,* and *Pandanus*), **e** Poaceae pollen grains, and **f** influx of charcoal particles (>160 µm). The vertical bars represent the five key periods of different paleoenvironmental conditions defined in the text.

A robust chronology was established for the sedimentary record of Lac Maudit by using a set of 19 ^14^C ages with an age-modeling approach (Supplementary section 1.2). For the environmental reconstruction, a multiproxy approach was applied, which combined palynological (and charcoal counting), granulometric and inorganic geochemical (X-ray fluorescence core scan, XRF) analyses (see Paleoenvironmental analyses in Methods). Based on pollen zones, granulometric properties as well as a Principal Component Analysis (PCA) of the XRF data, our results unveil five distinct periods of different climatic conditions associated with different plant assemblages (Fig. 2 and Supplementary section 1.1–1.4): a first period (25–15.2 kyr) with cold and dry conditions, a second and third period (15.2–11.8 and 11.8–5.5 kyr) related to increased humidity and temperature during the AHP, a fourth period (5.5–0.9 kyr) characterized by precipitation decline after the AHP, and a fifth period (>0.9 kyr) related to the increase of fire activity.

Prior to 15.2 kyr (period 1; 25–15.2 kyr), pollen assemblages show that montane vegetation (25–70%; Fig. 2c) and evergreen humid forest (10–40%; Fig. 2d) were growing together in Montagne d’Ambre. Today, the NP (>800 m asl) harbors only evergreen humid forest up to the mountaintop (1475 m asl). The montane vegetation that grows elsewhere in northern Madagascar above 1800 m asl under lower temperature and precipitation is absent in Montagne d’Ambre (Fig. 1a and see Environmental settings in Methods). Therefore, the occurrence of montane vegetation in Montagne d’Ambre prior to 15.2 kyr suggests drier and cooler climate conditions than in modern times, similar to what was described for tropical East Africa^27,28^. Dry conditions at the study site are also evidenced by low sediment-accumulation rates of ca. 0.07 mm a^−1^ revealed by the age model (Fig. S2). Furthermore, texture and grain size show a very compact sediment with silty-to-clayey deposits (mean grain size of 7.5 µm) related to reduced precipitation and runoff amounts^29^ (Fig. 2b and Supplementary section 1.1). The PCA carried out with XRF data resulted in three main axes, with Axis 1 representing 61.5%, Axis 2 24.4%, and Axis 3 9.4% of the total data variance (Supplementary section 1.3). The first axis shows negative loadings for all measured elements, which is interpreted as high minerogenic input and low organic matter content consistent with dry conditions during the first period (Fig. S3). For the second axis, positive (negative) values mainly reflect increase (decrease) of iron (Fe), titanium (Ti), and nickel (Ni) that correspond to an enhanced (reduced) chemical weathering related to more (less) humid conditions and associated forest soil formation (Fig. 2a and Supplementary section 1.3). Increased siliciclastic input (high loadings for silicon (Si), calcium (Ca), and strontium (Sr); Fig. S3) into the lake prior to 15.2 ka, reflected by low values in Axis 2 (Fig. 2a), points to dry environmental conditions favoring physical weathering of bare, exposed materials (bare rock)^30^. Due to the dry environmental conditions, a low soil-formation rate and development of montane vegetation was favored, associated with enhanced physical weathering. During period 1, we therefore conclude that montane vegetation was present in the upper parts of Montagne d’Ambre at lower altitudes than is observed today in other regions of northern Madagascar. The evergreen humid forest also shifted to lower elevations in comparison with modern times and/or was restricted to sheltered habitats with humid local conditions in Montagne d’Ambre (e.g., valleys, rivers). However, a wide lowland expansion of this forest would be unexpected due to the rather dry conditions recorded between 25 and 15 kyr in tropical East Africa^26^, which were accompanied by a savanna expansion in African lowlands^31^.

During the second period (15.2–11.8 kyr), an increase in sediment accumulation rate by one order of magnitude (mean ca. 0.6 mm a^−1^; Fig. S2) along with larger, but variable mean particle sizes (6–23 µm; Fig. 2b) is pointing to higher transport energies and generally more erosive processes in the catchment. A sudden sediment color change from gray to dark-brown likely indicates higher amounts of organic matter and thus a stronger bioproductivity (Supplementary section 1.1 and Fig. S1). Increasing values in Axis 2 of the PCA show a trend toward higher portions of Fe, Ti, and Ni within the deposits (Fig. 2a and Supplementary section 1.3), which is interpreted as enhanced chemical weathering and deep soil formation. Contemporaneously, a rapid development of evergreen humid forest taxa (>50%; Fig. 2d) at the expense of montane vegetation (<25%; Fig. 2c) is observed. From these proxies, we infer a spectacular increase in moisture availability and thus enhanced precipitation in Montagne d’ Ambre. The major increase of evergreen humid forest also indicates an upward shift of the altitudinal vegetation zones and suggests a temperature increase in addition to precipitation increase. During this period, a short increase of fire events (14.5–13.5 kyr; Fig. 2f) is indicated by charcoal influx. However, the low values of charcoal influx (less than three particles per cubic-centimeter sample) do not support local fire occurrences at the catchment of the lake. Instead, this may rather suggest a development of vegetation prone to fire in the region, supported by an increase of *Myrica* pollen (Fig. S4). A likely increase of thunderstorms with lightning frequency and the development of suitable combustible biomass may best explain the detected episodes of wildland fires. Starting at 15.2 kyr, period 2 matches perfectly with the onset of the AHP documented in Madagascar’s central highlands^32^ and in East Africa^26^. This shows that climate changes were synchronous across this entire region.

Maximum proportions of evergreen humid forest (>60%; Fig. 2d) start at 11.8 kyr and indicate a third period (11.8–5.5 kyr) at the transition to the Holocene. From 10 to 5.5 kyr, even enhanced chemical weathering is evidenced by maximum values in Axis 2 of the PCA performed on XRF data (Fig. 2a) and rather fine-grained, dominantly silty and clayey deposits, comparable with those from period one (mean grain size ca. 7 µm), are observed (Fig. 2b). This likely points to maximum rates of precipitation within the entire record and to a quite high lake level. The latter is further supported by low abundance of aquatic plants (<5%; Fig. S4), which are generally related to shallow lake conditions. We conclude that during this period, evergreen humid forest grew on the entire mountaintop as it is observed today. In addition, the maximum rates of precipitation recorded during this period imply a lower altitudinal limit and thereby wider distribution of this forest type than today.

During the fourth period (5.5–0.9 kyr), a coarsening in the particle size (>10 µm; Fig. 2b) as well as higher abundance of aquatic plants (10–30%; Fig. S4) suggest a lake level drop, likely indicating generally drier conditions associated with reduced precipitation. A contemporaneous weakening in chemical weathering derived from lower values in Axis 2 (PCA of XRF data; Fig. 2a) is also consistent with a reduced moisture availability. These results denote the termination of the AHP and match with paleoecological data from East Africa where the termination of the AHP starts around 5 kyr^26^. In Montagne d’Ambre, this precipitation decrease may have led to a contraction of the evergreen humid forest by moving upward its lower altitudinal limit as also observed in East Africa^33^. However, with its central and higher location in Montagne d’Ambre, the lake site was probably not able to record this vegetation shift. This certainly explains why, except for a few changes in forest composition after 5.5 kyr, the evergreen humid forest surrounding the lake has not been markedly impacted by this reduction of humidity (Fig. 2d and Supplementary section 1.4).

The last ~0.9 kyr, corresponding to the fifth period, are characterized by reduced evergreen humid forest taxa (ca. 40%; Fig. 2d), although still present at Lac Maudit. This period is marked by a tremendous increase of charcoal particles reflecting an important increase in fire frequency (Fig. 2f). Combined with the high amounts of Poaceae pollen (>40%; Fig. 2e), this suggests a major vegetation change. Today, most fires occur in the surrounding lowlands (>6–7 km away) dominated by open grasslands that are strongly affected by human activities through slash-and-burn agriculture and extensive cattle grazing. While evergreen humid forest in Montagne d’Ambre prevents fire ignitions, the permanent occurrence of charcoal particles recorded since 0.9 kyr with recurrent peaks exceeding 20 particles per cubic-centimeter sample is certainly the result of an important shift of lowland ecosystems to an open grassland ecosystem associated with high fire frequency. Charcoal particles produced by these fires have been certainly carried up by aeolian transport to Lac Maudit. The high amount of Poaceae pollen, probably transported by the same process, also supports the occurrence of an open-grassland ecosystem in the lowlands. The contemporaneous development of swampy vegetation, harboring Poaceae plants, at the lake site (Supplementary section 1.4) may have also contributed to the elevated amount of Poaceae pollen. While human arrival during the early or mid-Holocene is still hotly debated for Madagascar^34,35^, it has been shown that humans impacted northwestern Madagascar, only a couple of hundred kilometers south of Montagne d’Ambre, significantly through a rapid transition to a grass-dominated landscape with an abrupt increase in fire activity at ~1.2 kyr^36,37^. Furthermore, large settlements developed from 1 kyr along the coast such as at Lakaton’i Anja located next to Montagne d’Ambre^38,39^. In congruence with these findings, our results suggest an important human impact on the northern landscapes at least over the last millennium.

### Late quaternary lemur population dynamics

**Fig. 3:**
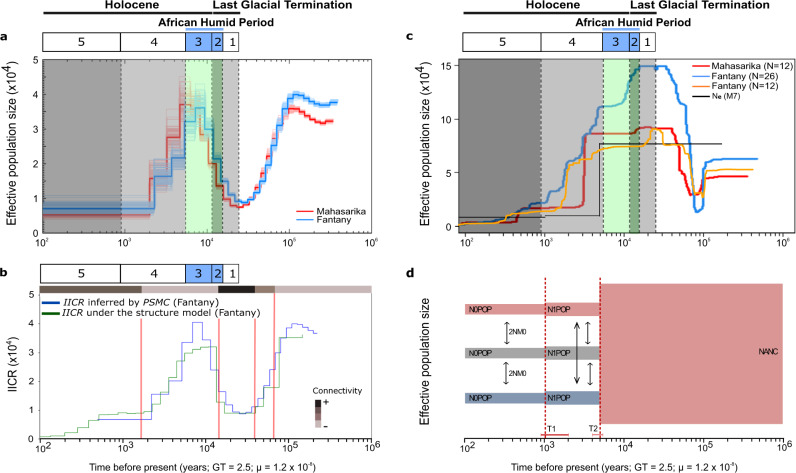
Integration of the four complementary demographic approaches used in this study. **a** Inference of the demographic history of *M. arnholdi* with the *PSMC* method. The thick red and blue lines represent the inferred trajectories of the northern (Mahasarika) and the southern (Fantany) site. Each light line represents 100 subsampled bootstrap replicates for each individual. The legend on the top identifies the five periods of environmental change derived from the paleoenvironmental reconstructions defined in the text. **b** *IICR* inferred by the *PSMC* method for Fantany (blue) and *IICR* under an n-island model of migration considering a constant population size and four changes in population connectivity (green). Vertical red lines mark the times of change in connectivity (~65, ~40, ~10−15 and ~1–2 kyr). Horizontal bars on top in gray shades indicate relative levels of connectivity (see Supplementary section 2.1.9 and Fig. S6 for details about the connectivity inferences). **c** Demographic history inferred for Mahasarika (red, *N* = 12) and Fantany (blue, *N* = 26) with the *Stairway Plot* method. The demographic dynamics of Fantany were repeated considering an equal sample size as for Mahasarika (orange, *N* = 12). For reasons of direct comparison, the black line illustrates the effective population-size dynamics as suggested by the best-fitting demographic model (M7) by *fastsimcoal2* over time. **d** Illustration of the best demographic model (M7) revealed by *fastsimcoal2*. The model suggests the occurrence of two consecutive population declines during the last 5 kyr. The different populations are represented by distinct colors. An additional population (“ghost population”) was included to represent intermittent *M. arnholdi* sampling sites that were not covered by our sampling scheme. The width of bars is proportional to the estimated effective population size (*N*_*e*_, details in Table 1). The occurrence of gene flow is exemplified by arrows. N0POP = effective population size for each population at present time; N1POP = effective population size for each population after the first population decline; NANC = ancestral population size; 2NM0 = average number of haploid immigrants entering the population per generation. T1 = time of the younger population decline; T2 = time of the older population decline. Horizontal error margins represent the 95% confidence intervals for the time of the population declines. Parameter estimates are summarized in Table 1. All analyses were performed considering 2.5 years as generation time. See also Supplementary section 2.2.4 and Figs. S13 – S16 for demographic inferences considering a generation time of 1.0 and 4.5 years.

The four complementary demographic approaches implemented in this study confirm that *M. arnholdi* underwent substantial demographic changes during the past 25 kyr. The *Stairway Plot*^21^ and the Pairwise Sequentially Markovian Coalescent (*PSMC*)^19^ methods were consistent in inferring the occurrence of an apparent population decrease in recent times (periods 5 and 4, <5.5 kyr) in both Mahasarika and Fantany (Fig. 3a,c). This population decrease was preceded by a period of a large population size, itself preceded by a more ancient population bottleneck. While the two methods identified a similar overall trend, the temporal dynamics were different among them. The ancient population bottleneck inferred by the *Stairway Plot* (Fig. 3c) dated >25 kyr, whereas the *PSMC* method (Fig. 3a) suggested lowest population sizes at the beginning of period 1 (25–15.2 kyr). Although the methods differ in the duration of the subsequent population recovery, both methods agreed that mouse lemurs had a relatively large population size during the maximum humid conditions of the AHP (i.e., period 3, 11.8–5.5 kyr) at both sampling sites. Differences on the temporal dynamics among the two methods are not surprising, as it has been shown that *PSMC* performs best for ancient times, while the *Stairway Plot* tends to perform best toward the present^40–43^. However, the interpretations of the previous demographic approaches rely on the assumption that population structure can be neglected and that mating is random. In an attempt to overcome this limitation of panmictic-based interpretations of the *PSMC*^44–46^, we simulated the Inverse Instantaneous Coalescent Rate (*IICR*)^47^ of the southern site (Fig. 3b) by changing connectivity parameters, but assuming a constant population size. The model parameters that generated the best fit between the *IICR* in such a structured model and the dynamics inferred by the *PSMC* method suggest an n-island model of migration (29 demes interconnected by symmetrical gene flow) with four changes in connectivity. Those changes in connectivity dated around 65, 40, 10–15 and 1–2 kyr (vertical lines in Fig. 3b). More specifically, the *IICR* simulations suggest a period of high connectivity that started long before period 1 (~40 kyr), which was followed by a period of reduced connectivity during the AHP (~13 kyr) (see Supplementary section 2.1.9 for details about connectivity inferences). Such a scenario is unlikely, since the paleoenvironmental records revealed relatively unfavorable conditions (=lower extension of evergreen humid forest) during period 1 (25–15.2 kyr) and increasingly favorable conditions during the AHP (= maximum extension of evergreen humid forest). We therefore conclude that population size changes explain the *PSMC* dynamics during this time period better than changes in population connectivity (but assuming no change in population size).

To disentangle the confounding effects of population-size changes and structure, and allow for both factors to potentially explain our genomic data, we built and compared the congruence of 13 alternative demographic models of increasing complexity (M1–M13) to the RADseq data using a composite likelihood framework implemented in *fastsimcoal2*^20^. The models incorporated various combinations of changes in population size, population structure (with models involving population splits), and changes in connectivity among the sampling sites (Supplementary Figs. S8 and S9). The likelihood comparison of all 13 demographic models revealed that models including population structure had a better fit than those assuming panmixia, but also that recently structured models (i.e., models where a panmictic population become recently structured) with population size changes showed the highest congruence to the observed data (Supplementary results 2.2.3 and Table S6). The best fitting model (M7) suggested the existence of a large ancient population that only became structured (i.e., split in several demes) and suffered a first reduction on population size around 5 kyr. This event was followed by a more recent population decline at ~1 kyr (Fig. 3d, Table 1). However, the high estimated levels of gene flow among the populations after they became structured (2NM0 ~7.23, where 2NM0 = average number of haploid immigrants entering the population per generation), suggest that the northern and southern site were probably never completely isolated from each other. This last approach not only allowed us to identify the most likely scenario incorporating both changes in population size and structure, but also demonstrates that both types of events are necessary to explain the data.

**Table 1:** Demographic parameters inferred under the best fitting demographic model (M7).

		95% CI	
Parameter	ML estimate	Lower bound	Upper bound
N0POP	**13,937**	11,796	14,133
N1POP	**14,329**	13,161	17,773
NANC	151,573	109,463	147,754
T1	**1,073**	910	2,025
T2	**4,893**	4,018	5,430
2NM0	**7.23**	6.73	9.34

Maximum-likelihood (ML) estimates were obtained from the run with the highest composite likelihood. The respective 95% confidence intervals (CI) were generated by block-bootstrapping. All population size estimates are given in number of haploid copies. Time changes were scaled considering GT = 2.5 years. N0POP = effective population size for each population after the most recent population decline; N1POP = effective population size for each population after the older population decline; NANC = ancestral population size; 2NM0 = average number of haploid immigrants entering the population per generation. T1 = time of the most recent population decline; T2 = time of the older population decline. See Fig. 3d for a schematic illustration of M7. In bold: ML estimates that lie within the 95% confidence intervals. Note that the NANC estimate should be treated with caution as it falls outside the 95% confidence interval range.

### Late quaternary paleoenvironmental shifts and first human impact on endemic fauna

Population genomics and paleoenvironmental reconstructions were generated from the same study area to investigate how past climatic changes impacted the population dynamics of a forest-dwelling species. One of the most striking results is the significant impact of the AHP on the paleoecology and on the demographic history of *M. arnholdi*. In particular, the *PSMC* inferred a population decline preceding the AHP (contemporaneous to the Last Glacial Maximum; period 1; 25–15.2 kyr), after which the abrupt precipitation increase of moisture conditions related to the AHP (period 2 and 3; 15.2–5.5 kyr) allowed a rapid and large development of evergreen humid forest across the entire mountain that triggered a population expansion of *M. arnholdi*. The alternative scenario assuming structured populations under constant population size (result of *IICR* simulations) can be rejected for *M. arnholdi*, since it predicted a lower connectivity during the AHP, which is unlikely, given the large expansion of evergreen humid forest during this time period. In particular, during period 3 (11.8–5.5 kyr), climate conditions supported an even wider extent of evergreen humid forest than in present times, which likely allowed mouse lemur populations to expand and grow further. Although we cannot determine the exact distribution limits of this forest during the AHP, specifically in lowland areas, the current occurrence of *M. arnholdi* in isolated humid-habitat patches outside the present limits of Montagne d’Ambre^24^ suggests a temporarily broad expansion of evergreen humid forest in the past. Connectivity between lemur populations should have been high during such periods of increased humidity, as it could have been the case during the AHP. This hypothesis is further supported by the discovery of subfossil lemurs, currently restricted to humid forests in eastern Madagascar (i.e., *Propithecus diadema, Indri indri*, and *Hapalemur simus*)^48–50^, in caves located in the currently dry forests of Ankarana, close to our study area (~25 km south of Fantany). Conversely, it is plausible that Montagne d’Ambre acted as a refugium for rainforest-adapted species during dry periods and facilitated recolonizations during subsequent humid periods^8,51^. Our interdisciplinary study revealed a strong mid-Holocene population decline of *M. arnholdi*, which occurred under precipitation decrease related to the termination of the AHP and thereby long before important human impact started in Madagascar^52^. Such a mid-Holocene population decline was also reported in multiple Malagasy taxa, including lemurs^12^, frogs^53^, endemic Eagle species^54^, and in the Malagasy giant rodents^55^. The first population decline was followed by a second decline in *M. arnholdi* at the beginning of the last millennium, which again parallels previously documented population bottlenecks for other lemur species^17,56,57^, all attributed to human-induced changes in forest habitats. Based on local paleoenvironmental reconstructions, we demonstrate that the latest population decline of *M. arnholdi* indeed coincided with a large increase of fire activity accompanied by a distinct ecosystem shift to a grassland-dominated landscape in the lowland areas surrounding Montagne d’Ambre. These changes most likely resulted from a regional increase in human activities and a shift to agro-pastoralism practices^58^. Although natural climatic changes might have also been relevant for ecosystem shifts in lowland areas during the past millennium, our results imply that human impact was certainly one of the dominant drivers in these latest and strong ecosystem changes in northern Madagascar.

## Conclusion

Our dual paleoecological–genomic approach demonstrates signatures of both past natural climate change and strong recent anthropogenic impact in northern Madagascar. While demographic dynamics of *M. arnholdi* were exclusively shaped by natural climate changes until the late Holocene, the dynamics during the last millennium were also shaped by anthropogenic impact. In particular, we argue that the population decline of *M. arnholdi* at 5 kyr was climatically driven by the termination of the AHP, but that human occupation combined with the increasing aridification after the AHP impacted this species even further. Therefore, the termination of the AHP had severe impacts also in northern Madagascar and potentially on large parts of the island, leading to substantial population fluctuations and declines in Malagasy wildlife prior to human impact^34,35^. This interdisciplinary study reveals that understanding extant biodiversity requires a critical knowledge of natural, climatically driven environmental and ecological changes during the past and particularly beyond the late Holocene when significant human impact started on the island. Additionally, this study illustrates that it is extremely valuable to use multidisciplinary datasets to validate demographic approaches and explore both population size and connectivity changes across different time scales.

## Methods

### Environmental settings

Montagne d’Ambre NP comprises an isolated volcanic massif in northern Madagascar that stretches 30 km from north to south and 15 km from east to west with an altitude range of 400–1475 m asl (Fig. 1a). The location close to the coast (Indian Ocean/Mozambique Channel) with frequent trade winds triggers high orographic rainfalls. This process, frequently observed in tropical mountains, is mainly explained by the altitude increase that cools the air causing frequent fogs and high orographic precipitation^59^. This evergreen humid forest is in clear contrast to drier conditions in the seasonally dry forests or open grasslands growing below 800 m asl around the mountain and the montane vegetation growing above 1800 m asl in other parts of the island^60,61^. In Montagne d’Ambre, only rare plants of montane vegetation currently occur in the upper-mountain parts. Consequently, there is currently no mountain vegetation zone in Montagne d’Ambre. Inhabiting the evergreen humid forests in northern Madagascar, the tree-dwelling primate *M. arnholdi* occurs in Montagne d’Ambre and its distribution extends to several highland humid forests between the Loky and Fanambana Rivers, southeast of Montagne d’Ambre^24^. A single *M. arnholdi* individual has also been reported close to Ambanja, southwest of the NP^62^.

### Coring

Lac Maudit (−12.582°N, 49.150°E, 1250 m asl) is characterized by a coverage of ca. 75% with floating vegetation and a peat bog. Open water still is present in the northern and southern part of the crater. From the center of the lake, accessible from the peat bog, two parallel sediment cores (LM1A and LM1B) with lengths of 10.5 m and 10.75 m were recovered using a Russian peat corer in June 2017. After coring, all 50-cm segments were described lithologically and subsequently wrapped in polyvinyldene chloride film. Cores were stored at 5 °C and under dark conditions until further work.

### Paleoenvironmental analyses

From both cores high-resolution, semi-quantitative measurements of inorganic chemical elements were conducted using an ITRAX (CS-8) X-Ray fluorescence (XRF) core scanner with a molybdenum (Mo) tube^63^ at the Geomorphological–Sedimentological Laboratory of the Geomorphology and Polar Research (GEOPOLAR), University of Bremen. XRF scanning was conducted at 55 kV and 30 mA with 10 s of exposure time at 0.2 mm resolution. Further, radiographic images were acquired with 40 kV, 15 mA, and 200 ms of exposure time at the same spatial resolution. Silicium (Si), potassium (K), calcium (Ca), titanium (Ti), manganese (Mn), iron (Fe), nickel (Ni), and strontium (Sr) showed reliable signals and were used for further interpretation. These elements were normalized to the counts of incoherent radiation (“Mo inc”) derived from the XRF scanner, to account for lithological changes and sediment matrix effects. Based on the lithological description (marker layers), the XRF-element patterns, and digital and radiographic images, LM1A and LM1B were parallelized and combined to a composite core. Using the XRF data, a Principal Component Analysis (PCA) was carried out, using the selected elements (Si, K, Ca, Ti, Mn, Fe, and Ni) as input variables. Prior to this multivariate statistic, the normalized elements were transformed using standard “z-transformation”. Grain-size distribution measurements were realized on LM1A, while paleoecological analyses, including pollen and charcoal counting, were realized on LM1B. For the grain size analysis ~1 cm^3^ subsamples at an interval of 5 cm were taken and measured after destroying carbonates and organic matter according to standard protocols with HCl (15, 30% p.A.) resp. H_2_O_2_ (10, 32% p.A.). Measurements were carried out with a laser diffraction particle-size analyzer (LS 13320 Beckman Coulter) in seven cycles of 60 s each. The first reproducible signal was considered as reliable and final distribution data were calculated using the Fraunhofer optical model. Statistical parameters were derived from the raw data using a modified version of the MS-Excel macro Gradistat (V.4.5 s)^64^. For pollen extraction, ~0.5 cm^3^ subsamples at an interval varying between 8 and 48 cm on LM1B were prepared following a standard chemical protocol. A minimum sum of 300 terrestrial pollen grains was counted for each subsample using a light microscope at 400x magnification. Pollen and fern-spore percentages were calculated on the terrestrial pollen sum. For charcoal-particle extraction, 1 cm^3^ of sediment was sampled every cm along the core LM1B and soaked in a 3% NaP_2_O_4_ solution plus bleach for several hours to deflocculate sediments and oxidize organic matter. Samples were sieved through a 160 μm mesh and charcoal particles were counted using a stereomicroscope at x40 magnification coupled to a digital camera. Influx of charcoal particles was calculated using the age-depth model (number of particles/cm^2^/year). For a reliable and robust chronology, 19 radiocarbon measurements were obtained by Accelerator Mass Spectrometry radiocarbon analyses from bulk organic matter of 1 cm^3^ sediment samples (Supplementary Table S1). All subsamples for radiocarbon dating were measured at Poznan Radiocarbon Laboratory (Poland) and are calibrated to calendar kiloyears before present (kyr) using the southern hemisphere terrestrial calibration curve SHCal13^65^. For more details see Supplementary sections 1.1−1.4.

### Sample collection of *M. arnholdi*

Mouse lemurs were sampled in the evergreen humid forest at the northern (Mahasarika, −12.534329 °N, 49.176295 °E) and southern limit of Montagne d’Ambre NP (Fantany, −12.695739 °N, 49.167458°E; Fig. 1a). The first site lies at 1073 m asl, while the second (approx. 18 km apart from Mahasarika) is located at slightly lower altitude at the ecotone between forest and grassland at 848 m asl. Field work took place between August and October 2017 (dry season). Mouse lemurs were captured by hand near the trees that they used during nighttime activity^66^ (see Supplementary section 2.1.1 for details). A total of 46 mouse lemur samples were obtained for genomic analyses, which included 14 M. *arnholdi* from Mahasarika and 32 from Fantany. All capture-routine protocols were approved by the Malagasy Authorities and by the Institute of Zoology, University of Veterinary Medicine Hannover (Research permit N°78/17/MEEF/SG/DGF/DSAP/SCB.Re). A sample-exportation permit for ear biopsies was granted by the authorities in Madagascar (permit number 855C-EA10-MG17) and an importation permit was granted by the Bundesamt für Naturschutz, Germany (permit number DE-E-06530/17).

### DNA extraction, RADseq, and whole-genome sequencing

Total genomic DNA was extracted from ear biopsies using the DNeasy Blood & Tissue Kit (Qiagen) following the manufacturer’s protocol with few modifications^67^. Two genomic datasets were generated to reconstruct the demographic history of *M. arnholdi*: a restriction site-associated DNA sequencing (RADseq) dataset and whole-genome sequences. Library preparation and RAD sequencing of all 46 samples were conducted at the GeT-PlaGe platform (Toulouse, France). In parallel, two mouse lemur samples (one from Mahasarika and one from Fantany) were selected for whole-genome sequencing (Supplementary Table S2). Both samples were derived from females in order to avoid a bias due to potential sex differences in dispersal behavior, which is common in other mouse lemur species^68,69^. The raw genomic data were trimmed and aligned to the *Microcebus murinus* reference genome^70^. All analyses using the RADseq dataset were performed based on genotype likelihoods^71–73^, considering only individuals with a mean sequencing coverage >4X^73–75^. ANGSD^73^ was used to call genotype likelihoods for sites present in at least 75% of the individuals, and with a base quality above 20, a mapping quality above 30, and with a minimum minor allele frequency above 0.05. For the analyses with the whole-genome sequences, we applied a missing data filter of 30% and controlled for read depth and quality of the variant sites by considering a base quality above 20, a mapping quality above 30, a minimum read depth of three, and a maximum read depth of 100 (see Supplementary sections 2.1.2−2.1.4 for further methodological details and 2.2.1 and Table S3 for details about the genomic datasets).

### Relatedness analyses and detection of population structure

In order to overcome a potential demographic history reconstruction bias^44,45,76^, the relatedness between the 46 sampled mouse lemurs was investigated first. The relatedness between two individuals is usually described by the concept of identity-by-descent (IBD). The software NGSrelate^74^ implemented in ANGSD framework^73^ was used to calculate the IBD coefficients (*k*_0_, *k*_1_, and *k*_2_) between all pairs of individuals of our dataset (see Supplementary section 2.1.5 for details). Of each dyad of closely related individuals (e.g., parent–offspring or full siblings), only one partner was retained in the dataset. Subsequently, NGSadmix^72^ was used to investigate population structure of 38 non-related individuals (12 from Mahasarika and 26 from Fantany). NGSadmix uses a maximum-likelihood clustering method to assign individuals to a predefined number of populations (K) based on genotype likelihoods^72^. A total of 10 independent runs of NGSadmix were performed to assign all individuals to two (*K* = 2) or three (*K* = 3) ancestral populations, assuming that the existence of more than three populations is not realistic at our local scale. The most suitable value of *K* was determined with Clumpak^77^, following the Evanno method^78^. The population structure analyses support the existence of two genetic clusters with low levels of admixture between the two sites (Fig. 1b; Supplementary section 2.2.2 and Fig. S7). Consequently, the two sampling sites were considered as two independent demes in all downstream analyses.

### Generation time

Demographic analyses are known to be affected by generation time (GT)^79^. Different generation time values ranging from 1 to 4.5 years can be found in the literature on mouse lemurs^11,17,80,81^. A GT = 1.0 was widely used in early demographic studies^17,80^ and corresponds to the age at first reproduction. A longer generation time (3.0–4.5 years) has been estimated based on survival data for a population of *M. rufus* in eastern Madagascar^11^. Recently, an intermediate GT value of 2.5 years was estimated for a free‐living *M. murinus* population in northwestern Madagascar based on parentage data^81^, where the authors defined GT as the average age of parents. A GT = 2.5 seems biologically appropriate for mouse lemurs, because it considered the entire reproductive career of individuals and took into account age- and sex-related differences in reproductive performance^81^. This estimate was used to generate all genomic results presented in the main text. However, in order to evaluate the consequences of choosing different generation times, all demographic analyses were repeated using the three aforementioned GTs (1.0, 2.5, and 4.5 years) and the results are presented in the Supplementary section 2.2.4.

### *PSMC* analyses and *IICR*

*PSMC*^19^ analyses were performed on two whole-genome autosomal sequences considering a minimum read depth per site of 3 (-d3) and a maximum read depth per site of 100 (-D100). The upper limit of the TMRCA was set to 5 (-*t*5) and the initial θ/ρ value to 5 (-*r*5). The *N*_e_ was inferred using a total of 100 bootstrap replicates. *PSMC* plots were scaled using generation times of 1.0, 2.5, and 4.5 years per generation (see Supplementary section 2.2.4 and Fig. S15), and a mutation rate of 1.2 × 10^−8^^11,82,83^. This mutation rate is the most accurate estimate available for mouse lemurs and was calculated from average pedigree-based estimates of seven primate species, including that of one mouse lemur species^83,84^. To evaluate the impact of the relatively low genome-wide coverage in our *PSMC* inferences, we also ran simulations for variant coverage divergence based on minimum read depth option per site (-d) between 1 and 9 for both study sites. The results are presented in the Supplementary section 2.2.5 and Fig. S18. Recent work has shown that *PSMC* dynamics can also result from changes in connectivity and not only from population size change, if populations are structured (e.g., *n*-island model, Supplementary section 2.1.6)^44,47^. It was shown that the *PSMC* method actually estimates a time- and sample-dependent function called the Inverse Instantaneous Coalescent Rate (*IICR)* whose interpretation is model-dependent (i.e., population panmixia vs population structure)^44^. The *IICR*^44,47^ can be predicted under a very large family of structured models using simulations to identify complex models that can replicate the observed *PSMC* curve. In order to investigate if the demographic trajectories revealed by the *PSMC* method could be explained exclusively by changes in population connectivity, the *IICR* of Fantany was computed considering an *n*-island model of migration with a constant population size. The model simulations were performed by varying the number of connectivity changes between two and four, the migration rate between 0 and 100 and considering the number of islands up to 100. Genomic data was simulated using the *ms* software^85^, and the *IICR* was computed and plotted with a python script available at: https://github.com/willyrv/IICREstimator^47^, assuming a mutation rate of 1.2 × 10^−8^ and the three generation times (see Supplementary section 2.2.4 and Fig. S16). The *IICR* obtained under the best structure model was finally compared to the one inferred by the *PSMC* method.

### Stairway *Plot*

The complete RADseq dataset of non-related animals (*N* = 38 individuals) was used for estimating a Site Frequency Spectrum (SFS; i.e., distribution of the allele frequencies of a given set of single nucleotide polymorphisms in a population)^16^. As a first step, ANGSD^73^ was used to calculate the site allele frequency likelihoods based on individual genotype likelihoods. The realSFS tool implemented in ANGSD^73^ was then used to estimate the 1d-SFS for each sampling site separately. Given that there is no suitable outgroup available to determine the ancestral state of each allele, we considered the minor allele-frequency spectrum (i.e., folded SFS)^18^ for all SFS-related analyses. The two-folded 1d-SFS  were finally used as input data for *Stairway Plot* v2.0^21^. Inferences were made based on 200 SFS for each sampling site, as suggested by the authors. The remaining 199 SFS were generated by bootstrapping, using the script provided by the software^21^. The plots were scaled using all three generation times (see Supplementary section 2.2.4 and Fig. S14) and 1.2 × 10^−8^ as mutation rate^11,82^. The analyses were performed considering the entire dataset for each forest site, and also an equal sample size for Mahasarika and Fantany (*N* = 12). For this analysis, the individuals from Fantany were randomly selected using R (R Development Core Team 2005).

### Demographic modeling with *fastsimcoal2*

A total of 13 potential and plausible demographic scenarios were designed for *M. arnholdi* and their composite likelihoods were compared following a hierarchical approach. First, five simple models, including a constant population size model, a single-population-size change model (expansion or bottleneck), an ancient or recent population structure model, and a connectivity change model, were explored (M1–M5; Supplementary sections 2.2.3, Fig. S8). Second, more complex demographic models were formulated based on the results of the previous step (Supplementary Table S6). Specifically, we compared four recently structured models (i.e., models where a panmictic population become recently structured) with the inclusion of one or two population bottlenecks after the populations become structured, or an ancient population expansion preceding that event (M6–M9; Supplementary Fig. S9a). Additionally, we also compared four models considering changes in connectivity among populations (with or without size change), where the changes in migration rate occurred in parallel to the population structuring or afterward (M10–M13; Supplementary Fig. S9b). See Supplementary section 2.2.3 and Table S6 for details of model selection. The analyses were performed with the RADseq dataset but, for computational reasons, only 20 individuals (i.e., 10 individuals from each sampling site) were selected for the 2d-SFS estimation. As a first step, ANGSD^73^ was used to calculate the allele-frequency likelihoods for each sampling site, and the realSFS tool implemented in the same program combined together with custom scripts was used to estimate the two-dimensional minor allele frequency spectrum (i.e., folded 2d-SFS^18^). It is important to emphasize that (i) Mahasarika is about 18 km apart from Fantany, (ii) mouse lemurs are known to disperse over very small distances (e.g., below 1 km for *M. murinus*)^86–90^, and (iii) our genetic structure analyses revealed only low levels of admixture among our study sites (Fig. 1b). For these reasons, an additional “ghost population” was included in our demographic models to control for the effect of an unsampled subpopulation on the estimation of migration rates between Mahasarika and Fantany (i.e., by representing a potential source of admixture between the northern and southern sites)^91,92^. The inclusion of such a “ghost population” is recommended when gene flow among the sampled populations is limited^92^, and it was shown to improve the fit of the demographic models in human populations^92^. F*astsimcoal2* v.2.6^20^ was subsequently used to compute expected SFSs from simulations under the 13 demographic scenarios (see Supplementary Table S4 for details about model parameters). The best demographic model was selected according to three criteria. The Akaike Information Criteria (AIC)^93^ was initially used to rank all 13 demographic models, and the models with the lowest ΔAIC (i.e., the models that minimized the distance between observed and expected SFS) were selected as the best ranked demographic models (Supplementary Table S6). Second, the fitting of the 2d-SFS obtained for the parameters that maximized the likelihood under model simulations were visually compared with the 2d-SFS estimated from the RADseq dataset^94^ (see Supplementary Fig. S11–S13). Third, a block-bootstrap approach was used to calculate 95% confidence intervals (CI) for the parameters estimated under the three best ranked demographic models^20^ (see Table 1 and Supplementary Table S5, S7, Fig. S10) and to select the best fitting demographic model (see Supplementary section 2.1.7 for the command line options). The *fastsimcoal2* scenario that best fitted the genomic data was finally scaled considering the generation times of 1.0, 2.5, and 4.5 years (Supplementary section 2.2.4 and Fig. S17). Knowing that *fastsimcoal2* provides information about the direction of population size instead of inferring the N_e_ through time, this shortfall was accounted by the implementation of the *PSMC* and the *Stairway Plot* methods.

### Impact of protein-coding sites in the demographic modeling of *M. arnholdi*

We reran the *PSMC*, *Stairway Plot*, and the best *fastsimcoal2* demographic model without protein-coding sites to exclude possible confounding effects of sites under selection. All the analyses were performed using the same command options aforementioned and considering 2.5 years as generation time. The results are discussed in the Supplementary section 2.2.6 and shown in Figs. S19–S21.

### Statistics and reproducibility

The Methods section and supplementary materials provide all details for statistical analyses. Specific details are also provided in figures when appropriate. All experiments and analyses performed in this paper are entirely reproducible.

### Reporting summary

Further information on research design is available in the Nature Research Reporting Summary linked to this article.

## Supplementary information

Supplementary Information

Description of Supplementary Files

Supplementary Data 1

Supplementary Data 2

Supplementary Data 3

Supplementary Data 4

Reporting Summary

## Data Availability

All RADseq sequences obtained in this study are publicly available at Sequence Read Archive (NCBI) in the BioProject PRJNA560399 (Number accession: SAMN14854044–SAMN14854081). Whole genome sequences are available in the BioProject PRJNA632451 (Biosample: SAMN14909740 for Mahasarika and SAMN14909741 for Fantany). The paleoenvironmental data are provided in the Supplementary Information (see supplementary data file 1–4).

## Code availability

Scripts used for all genomic analyses are available from the corresponding author on reasonable request.
