# Supplementary material for: Past environmental changes affected lemur population dynamics prior to human impact in Madagascar
Source: Commun Biol. 2021 Sep 15;4:1084. doi: 10.1038/s42003-021-02620-1 (PMC8443640; doi:10.1038/s42003-021-02620-1)
Supplement: Supplementary file 1 — Supplementary Information [file 42003_2021_2620_MOESM1_ESM.pdf]

Supplementary Information for

Past environmental changes affected lemur population dynamics prior to human impact in Madagascar

Helena Teixeira[1,a*], Vincent Montade[2,3,a], Jordi Salmona[4], Julia Metzger[5,6], Laurent Bremond[3], Thomas Kasper[7], Gerhard Daut[7], Sylvie Rouland[3], Sandratrinirainy Ranarilalatiana[8], Romule Rakotondravony[9,10], Lounès Chikhi[11,12], Hermann Behling[2,b], Ute Radespiel[1,b*]

[1] Institute of Zoology, University of Veterinary Medicine Hannover, Bünteweg 17, 30559 Hannover, Germany

[2] University of Goettingen, Department of Palynology and Climate Dynamics, Untere Karspüle 2, 37073 Göttingen, Germany

[3] ISEM, Université Montpellier, CNRS, EPHE, IRD, Place Eugène Bataillon, 34095 Montpellier, Cedex, France

[4] CNRS-UPS-IRD, UMR5174, Laboratoire Évolution & Diversité Biologique, Université Paul Sabatier, 118 route de Narbonne, 31062 Toulouse, France

[5] Institute of Animal Breeding and Genetics, University of Veterinary Medicine Hannover, Bünteweg 17p, 30559 Hannover Germany

[6] Veterinary Functional Genomics, Max Planck Institute for Molecular Genetics, Ihnestrasse 73, 14195 Berlin, Germany

[7] Friedrich-Schiller-University Jena, Department of Physical Geography, Loebdergraben 32, D-07743 Jena, Germany

[8] Université d'Antananarivo, Faculté des Sciences, Mention Biologie et Ecologie Végétale, Laboratoire de Palynologie Appliquée, B.P 905 - 101 Antananarivo, Madagascar

[9] Ecole Doctorale Ecosystèmes Naturels (EDEN), University of Mahajanga, 5 Rue Georges V - Immeuble KAKAL, Mahajanga Be, B.P. 652, Mahajanga 401, Madagascar

[10] Faculté des Sciences, de Technologies et de l'Environnement, University of Mahajanga, 5 Rue Georges V - Immeuble KAKAL, Mahajanga Be, B.P. 652, Mahajanga 401, Madagascar

[11] Instituto Gulbenkian de Ciência, Rua da Quinta Grande, 6, P-2780-156 Oeiras, Portugal

[12] Laboratoire Évolution & Diversité Biologique (EDB UMR 5174), Université de Toulouse Midi-Pyrénées, CNRS, IRD, UPS. 118 route de Narbonne, Bât. 4R1, 31062 Toulouse cedex 9, France

[a] These authors contributed equally

[b] These authors jointly supervised this work

* Corresponding author(s):

Ute Radespiel, Institute of Zoology, University of Veterinary Medicine Hannover, Buenteweg 17, 30559 Hannover, Germany, tel. +49-511-9538430.

Email: ute.radespiel@tiho-hannover.de

Helena Teixeira, Institute of Zoology, University of Veterinary Medicine Hannover, Buenteweg 17, 30559 Hannover, Germany, tel. +49-0511-9538429

Email: helena.teixeira@tiho-hannover.de

1. Supplementary information for the paleoenvironmental reconstructions

1.1. Sediment core description. The lithology of core LM1A and LM1B allows to distinguish three main stratigraphic units (Fig. S1). The first unit from the base of the core at 1,075 cm to 1,006 cm depth is composed of light brownish to grey sediments showing laminations of 0.5 to 1 cm thickness, intercalated with ~1 mm blackish layers. The latter are presumably composed of organic matter. XRF data reveal maximum values for silicone (Si), potassium (K), calcium (Ca) and strontium (Sr), although titanium (Ti), and iron (Fe), manganese (Mn) and nickel (Ni) show high values as well (Fig. S1). These silty to clayey deposits are very compact and are designated as highly minerogenic, organic-poor lacustrine sediments. From the second unit, sediment characteristics abruptly change at 1,006 until 208 cm core depth. In general, deposits are much coarser (silt increase, clay decrease), less compact, with brown to dark-brown color, likely indicating an enhanced organic carbon content. Enrichment in organic matter is further supported by the increase of the inc/coh ratio calculated from the XRF data, which can be used as a qualitative measure of organic matter[1,2]. Randomly intercalated grey to blackish thin layers (ca. 0.5 cm thick) are found and at the transition to this unit, and all elements recorded by the XRF scanner reveal significant minima. However, this second unit also shows high internal variability and can be divided in three sub-units (2a, 2b and 2c, Fig. S1). Between 1,006 and 665 cm (sub-unit 2a) an increased variability of particle size mainly controlled by the sandy and clayey fraction of sediment is observed. Si, K, Ca, Mn and Sr decrease or remain at low values, while Ti, Fe and Ni generally increase after 850 until 665 cm. Between 665 and 526 cm (sub-unit 2b), sediments are characterized by high clay contents before again low values were detected above 526 cm combined with an increased particle size variability. The XRF data are still characterized by high values of Ti, Fe and Ni. Between 526 and 208 cm (sub-unit 2c), these elements show higher variability combined with a general decreasing trend. In the sub-unit 2c, between 366 and 333 cm, sediments consist of coarse sand and reveal high values of Ca and Sr. Although the cause of this layer is slightly speculative, because of the occurrence of sand with gravels, we consider this layer as the result of a mass movement event, most likely originating from the steep crater wall at the western shore of the lake (on-shore failure scar with associated small fan). Then, within the uppermost 50 cm of this unit, sediment changes gradually in color to dark-brown to blackish. Gradually more plant macro remains were found, which mark the transition to the third unit starting from 208 cm to the surface, which is characterized as peat deposit. In this unit the entire set of XRF derived elements decrease rapidly to 0. From this pure organic unit, granulometric data were not obtained.

Based on lacustrine sedimentation conditions, the two lowermost units (1 and 2) can be easily differentiated by varying minerogenic input and grain size and are assumed to consist of significantly different amounts of organic matter (Fig. S1). Since Montagne d'Ambre is geologically quite homogenous[3], and Lac Maudit, located near the mountaintop, has a small catchment area of

only ca. 1 km², varying sediment origin seems rather unlikely to explain these differences between the two lowermost units. A more reasonable explanation of the observed differences between these two units is certainly a “dilution” effect of the minerogenic fraction due to an important increase in organic matter in the second unit[4]. In addition, as already described in other regions[5,6], distinct changes in vegetation composition may have also contributed to the observed differences between these two units (see 1.4). Deep soils in Montagne d’Ambre are partly formed by alteration of an old gneiss[3] which might be the source of Si, Ca, K and Sr. Distinctly moister environmental conditions in the second unit (after 15.2 kyr), associated with a much denser forest cover than in the first unit, are assumed to have led to a thicker soil formation and hence to steadily increasing input of e.g., Fe, Ni and Ti. Consequently, these main changes can be summarized as following: Unit 1 - low organic input, low influx of Ti, Fe, Ni, high influx of Ca, Sr, K, Si (presumed from erosion of physically weathered material), small particles and low sedimentation rate (see 1.2). Unit 2 - increase in amount of organic matter, increase of Fe, Ti and Ni inputs associated with coarser grains (presumed form soil formation processes due to warmer and moister conditions), thus decrease in Si, Ca, K, Sr, and higher sedimentation rate.

a

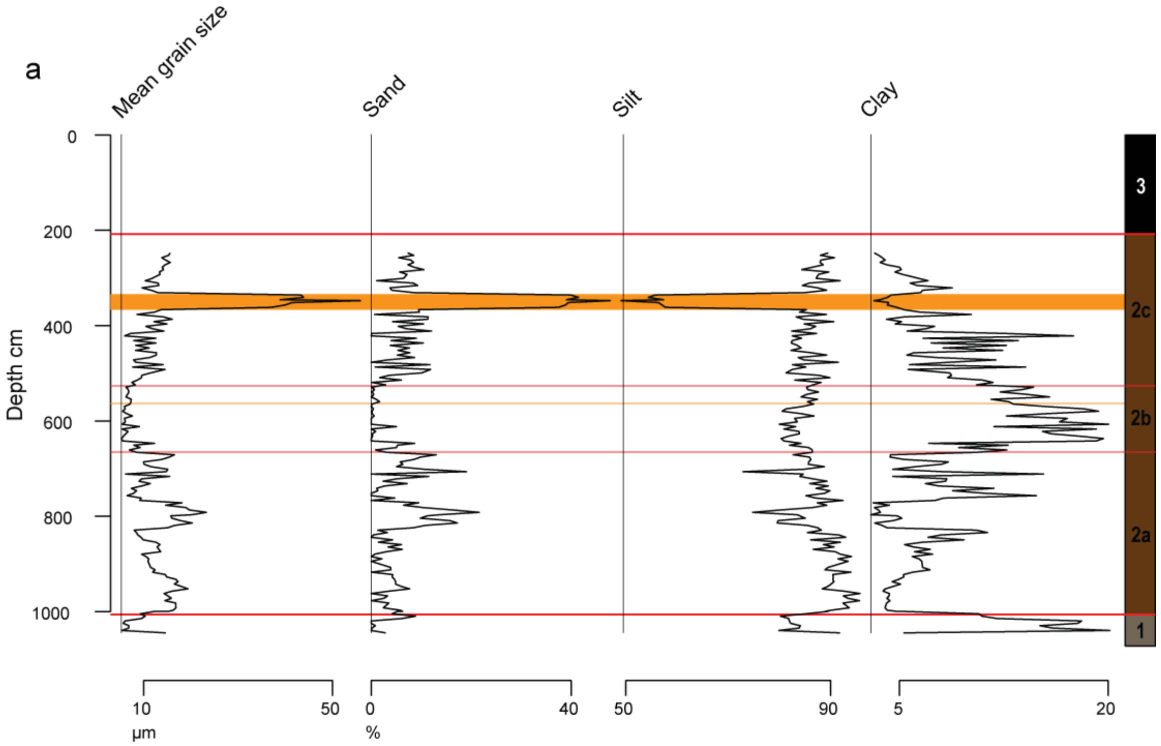

b

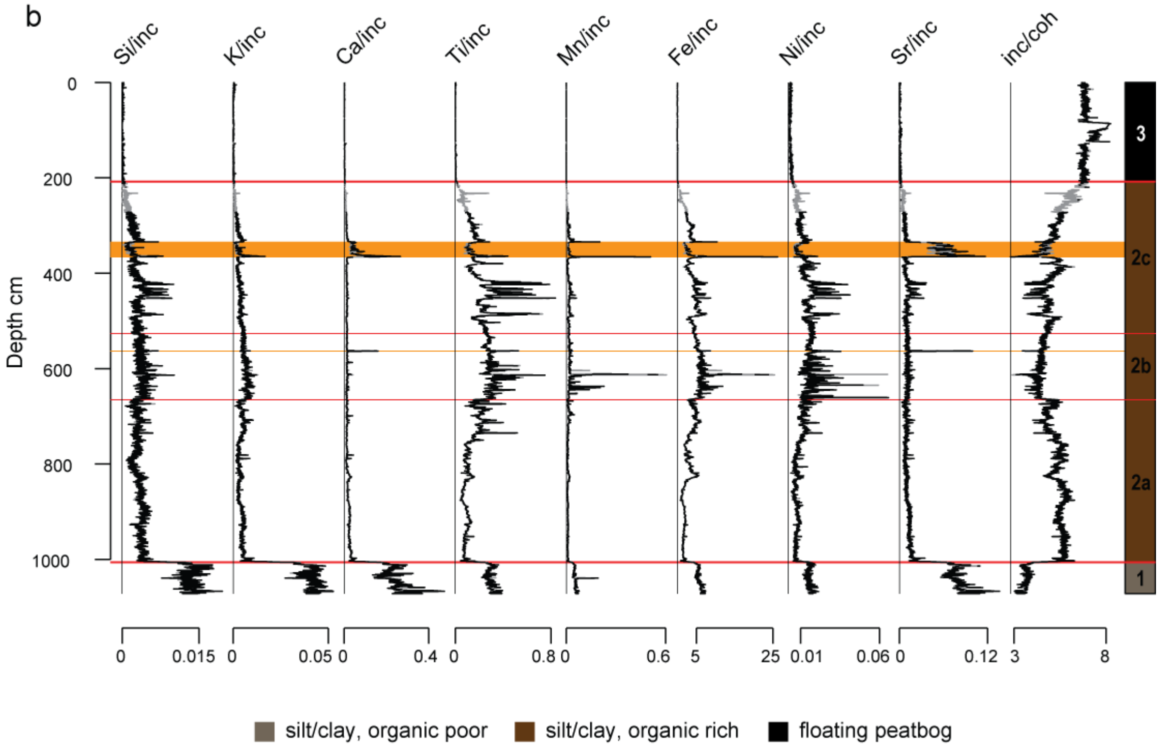

Fig. S1. Sedimentological description. a, grain size measurements performed on core LM1A. **b,** XRF scanning of the main elements and the ratio between the counts of the "incoherent radiation" and the "coherent radiation" (inc/coh). The elements were normalized to the counts of the

"incoherent radiation" (Mo-inc). XRF measurements performed on LM1A and LM1B were combined in one master core. Grey lines represent all data point measurements characterized by a mean square error >2. Grain size and XRF data are plotted on the same master core depth. The bar on the right side and the red lines represent the main sedimentological units and sub-units described in 1.1. The orange bars represent event-related deposits originating from the catchment that were removed to correct the master core depth before establishing the age-depth model (see 1.2).

1.2. Chronology. Based on the sedimentological description, layers in the second lithologic unit with high and abrupt increase of Ca were considered as event-related deposits originating from the catchment (Fig. S1). Two such events disturbed the normal sedimentation process in this unit: the 33 cm thick layer that we consider as the result of a mass movement event (366-333 cm; Fig. S1) and the 2 cm thick layer with a peak of Ca (564-562 cm; Fig. S1). Since these two layers are supposed to have been deposited within only hours (maybe days), they were removed to correct the master core depth before establishing the age-depth model. At ca. 234 cm we obtained an age of 505 ± 30 ^{14}C yr (Table S1). We therefore did not consider the peat bog sampled above the lacustrine sediment in the age-model for paleoenvironmental reconstructions. Concerning the two first units, we decided to build two separate age-models which we combined afterwards (Fig. S2). This option was selected because the smooth spline age-model (selected for the second unit) would have artificially increased the age of the transition between the two first units at 1,006 cm by smoothing the pronounced change in sedimentation rate, evidenced by lithology and confirmed by radiocarbon dating.

In the short first unit of 70 cm length, five Accelerator Mass Spectrometry (AMS) radiocarbon datings were obtained (Table S1). However, the lowermost ages reveal an inverse trend with ages getting progressively older towards the top of the section. Three ages appear to be contaminated by old carbon that might be the result of dissolution of old carbon with the weathering and erosion of deep soils (see 1.1) during that period, and the low carbon content of these datings may have contributed to the age uncertainties (Table S1). These three dates only show maximum ages and were therefore considered as outliers. Based on a linear interpolation, this unit covers a time span from 24.6 kyr to 15.2 kyr and is characterized by a very low sedimentation rate of ca. 0.07 mm a^{-1}. Because of high uncertainties revealed by the outliers, we only consider this unit as older than 15.2 kyr and environmental reconstructions from that first unit are considered as a snapshot of environmental conditions during the late Pleistocene prior to 15.2 kyr. In the second unit of 760 cm length, 14 AMS radiocarbon datings were obtained and a smooth spline age-model has been applied (Fig. S2 and Table S1). Between ca. 14.6 kyr and ca. 10 kyr, in the sub-unit 2a, characterized by a generally high sedimentation rate ranging between 0.3 and 1 mm a^{-1}, three outliers that could be also the result of dissolution of old organic carbon generate some uncertainties. Under increased humidity at the beginning of the African Humid Period (AHP)

evidenced by erosion and vegetation changes (see 1.3 and 1.4), a raising lake level is assumed. As already documented in other lakes[7] this process is often associated with reworking of older sediments or soils at the shore of the lake, which likely have resulted in the observed age inversion. After 10 kyr, the sedimentation rate in the sub-unit 2b and 2c remains at a constant range between 0.3 and 0.5 mm a^{-1}.

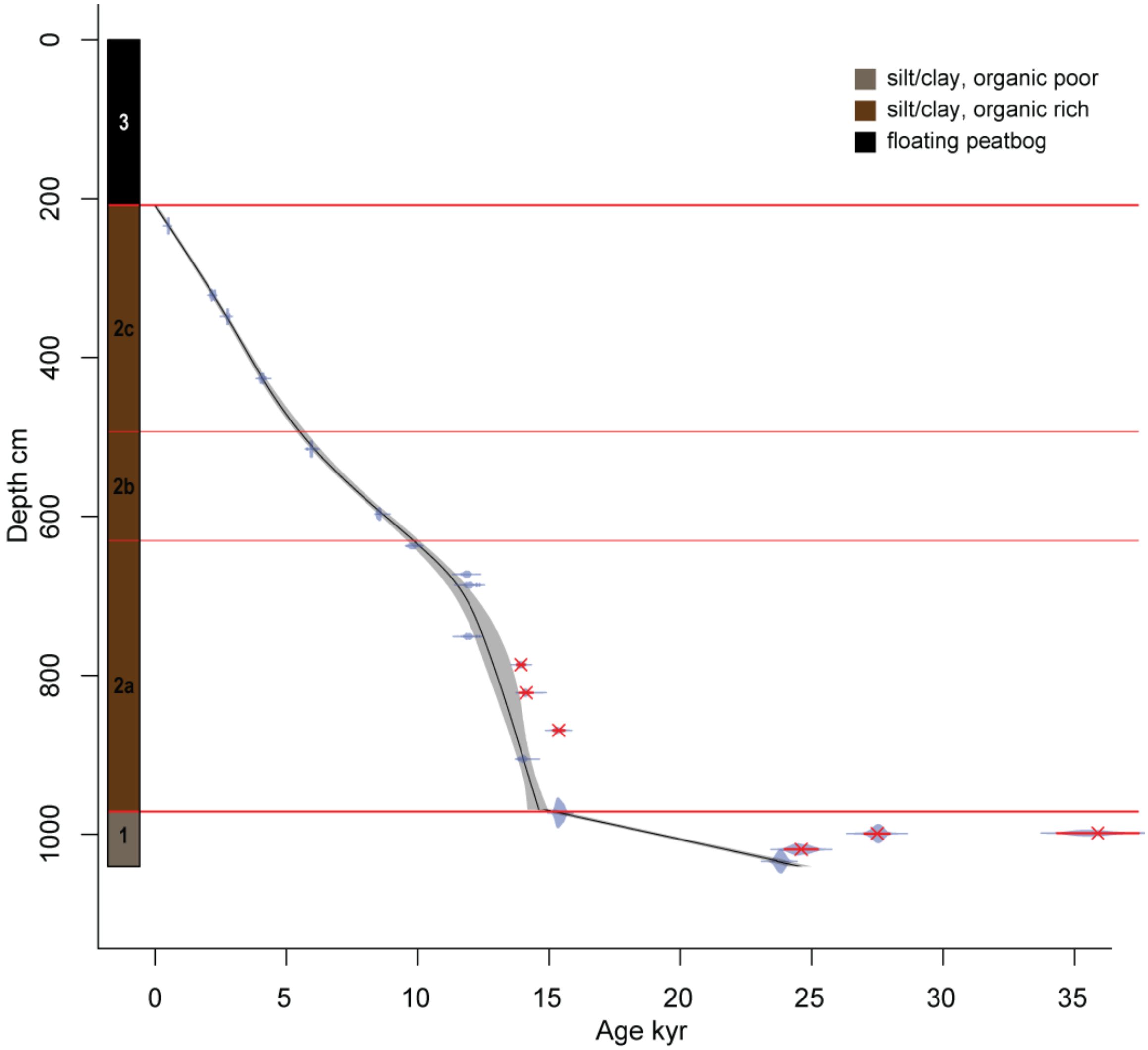

Fig. S2. Age-depth model of the composite master core. All the radiocarbon ages are calibrated to calendar kiloyear before present (kyr) using the southern hemisphere terrestrial calibration curve SHCal13[8]. Datings indicated in red represent the outliers removed before establishing the age-depth model. The bar on the left side and the red lines represent the main sedimentological units described in 1.1. The age-depth model was built running the package "Clam" (Version 2.2[9], with the RStudio software version 1.2.1335).

1.3. Principal Component Analysis with XRF data. In order to condense the information of the XRF core scan data, a Principal Component Analysis (PCA) was carried out, using the selected elements (Si, K, Ca, Ti, Mn, Fe, Ni) as input variables (Fig. S3). The PCA results in three main axes representing 61.5 % (Axis-1), 24.4 % (Axis-2) and 9.4 % (Axis-3) of the total data variance. Axis 1 shows high (negative) loadings for all measured elements which is interpreted as general minerogenic input. There is no anti-correlated member to this terrigenous input, however the down-core variability of this principal component suggests an "unknown" member, which we suspect is organic matter (either from primary aquatic production, from input of higher terrestrial plants, or from both). The XRF derived inc/coh ratio, which is influenced by an entire set of light elements (H, C, N, O) and is known to represent content of organic matter at least to some extent[1], shows a negative correlation to all elements, which supports our assumption. Sediment color, which changes from light gray-brownish to dark brown between the two first units when down-core axis 1 data also reveals a remarkable shift and further corroborates the assumption.

Axis-2 is characterized by two groups of elements with high positive loadings for Ti, Fe, and Ni and negative loadings for Si, K, Ca and Sr (Fig. S3a). These two groups reflect the difference between input of siliciclastic components (Si, Ca, Sr) from erosion of bare physically weathered material, and input of material supplied by soil formation processes (Fe, Ti, Ni). In the second unit after 15.2 kyr, a rising tendency until ~10 kyr followed by a decrease tendency after ~5.5 kyr indicate the shift towards more and less Ti, Ni and Fe inputs respectively (Fig. S3b). We therefore consider increase (decrease) of Ti, Ni and Fe as an enhanced (reduced) weathering related to more (less) humid conditions and associated rainforest soil formation.

Axis-3 is reflecting Mn which might indicate redox-conditions[10]. However, this seems to be "event-related" and might only occur during very strong short-term input of sediment (one major peak at ca. 612 cm which is right above the highest peak in Ti). However, Fe and Mn are also showing significant correlation ($r = 0.69$) suggesting that Fe is related to both, minerogenic input (erosion) and redox-conditions.

Mainly based on axes-1 and -2 we can thus synthesize the main sedimentological changes and erosion/weathering processes during the past 25 kyr. Prior to 15.2 kyr minimum values of axis-1 and -2 reflect low organic matter content in the sediment and influx of primarily physically weathered siliciclastic material. The strong and abrupt increase of axis-1 at 15.2 kyr indicates a "dilution" effect of the minerogenic fraction by an enrichment of organic carbon. After 15.2 kyr until ca. 10 kyr, a progressive increase of axis-2 suggest that environmental conditions steadily shifted towards more humid conditions favoring soil formation and chemical weathering. After reaching highest values between 10 and 8 kyr, axis-2 values stabilize until 5.5 kyr. Associated with an increased variability, the axis-2 values tend to decrease from 5.5 kyr onward, reflecting a progressively reduced humidity associated with reduced input of Fe, Ni and Ti. This is combined with an enrichment of organic carbon content as shown by increase of axis-1 values after 5.5 kyr.

In the uppermost part of the core, this previous trend strengthens showing the transition to the peat bog with high organic carbon content.

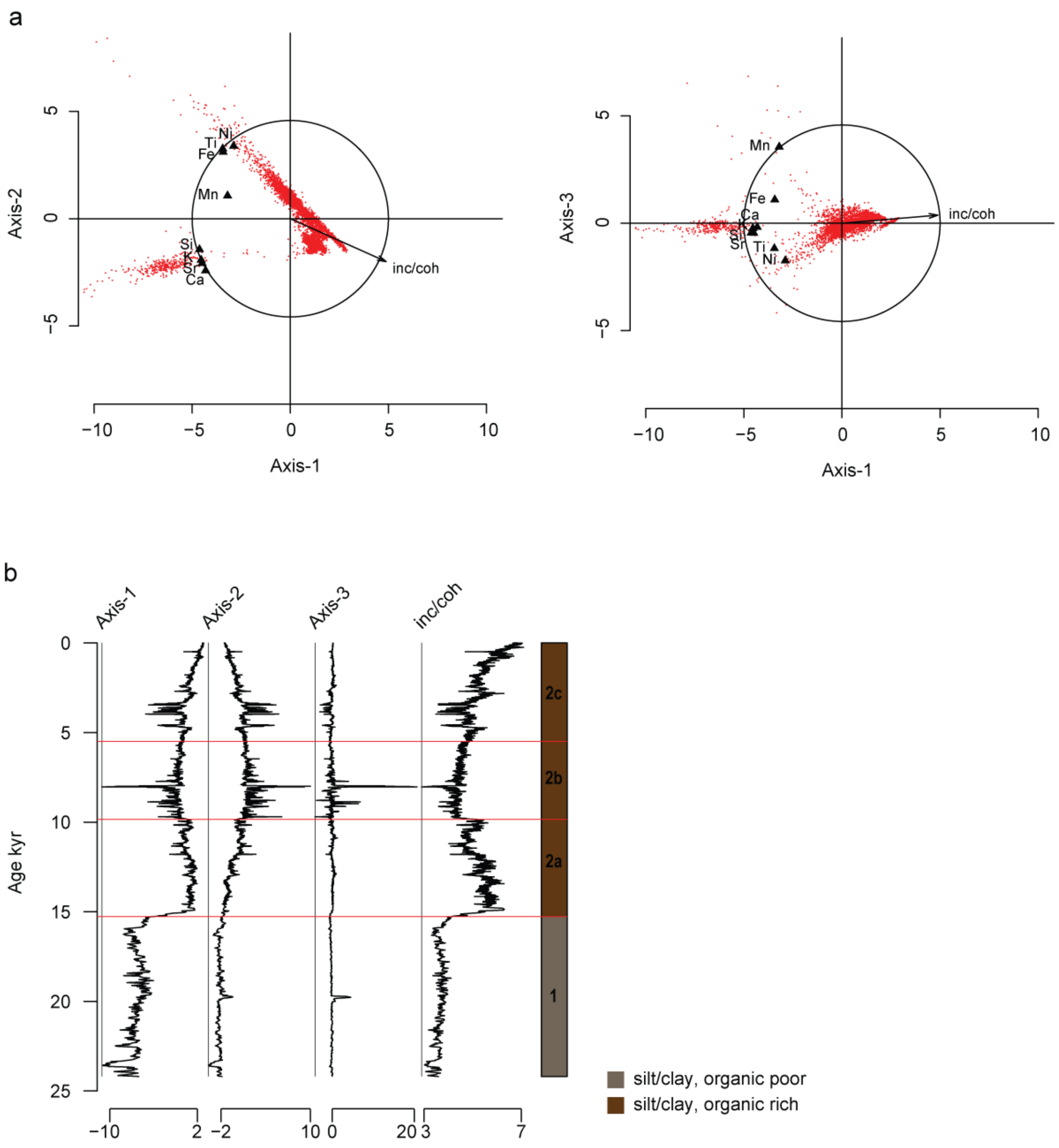

Fig. S3. Principal component analysis of XRF data of the composite master core. a, Bi-plot of Principal Component Analysis (PCA) for axis-1 and -2 and for axis-1 and -3 performed on the XRF data of the composite master core. Red dots indicate the distribution of samples and black triangle indicate the distribution of the selected elements to run the PCA. The ratio between the counts of the "incoherent radiation" and the "coherent radiation" (inc/coh) was passively projected on the axes of the PCA bi-plots. **b,** plot of PCA axes against the age scale indicated in calendar

kiloyear before present (kyr). The bar on the right side and the red lines represent the main sedimentological units described in 1.1. The PCA was performed using the RStudio software (Version 1.2.1335) running the package "Ade4" (Version 1.7-13)[11].

1.4. Pollen and charcoal data. The pollen and charcoal records from Lac Maudit allow to reconstruct vegetation history from Montagne d'Ambre during the past 25 kyr. A total of 206 pollen and spore taxa have been identified and the main pollen taxa have been summarized in Fig. S4. Pollen and spore identification were based on several atlases[12–15], the online African Pollen Database (11/2019 – http://apd.sedoo.fr/accueil.htm) and the reference collections from University of Goettingen (11/2019 – http://www.gdvh.uni-goettingen.de/) and from University of Montpellier (11/2019 – https://data.oreme.org/palyno/palyno_gallery). Based on the cluster analysis performed on terrestrial pollen grains, three main pollen zones (PZ1, PZ2 and PZ3) have been distinguished and compared with the lithology. PZ1 with subsamples prior 15.2 kyr is dominated by Ericaceae, *Podocarpus* and *Myrica* reflecting the occurrence of montane vegetation in Montagne d'Ambre. Today, this vegetation, including the lower montane forest and the montane shrubland, generally grows above 1,800 m a.s.l., such as in the Tsaratanana massif located 200 km south of Montagne d'Ambre[16]. With a maximum elevation of 1,475 m a.s.l., characteristic taxa of montane vegetation are not abundant in the modern vegetation of Montagne d'Ambre which is entirely dominated by evergreen humid forest from low and mid-altitude[17–19]. Montane vegetation developed before 15.2 kyr at Montagne d'Ambre, especially with a high amount of Ericaceae, which is characteristic for montane shrubland and hence suggests drier and colder climate conditions in comparison to modern conditions[16]. Moreover, in PZ1, high relative percentages of aquatic plants with *Potamogeton* or Cyperaceae are recorded. In particular, *Potamogeton*, a submerged leafed macrophyte typical for aquatic plants generally grows in lakes with large shallow areas[20]. Under dry conditions evidenced by forest composition, a shallow lake level could explain development of these taxa. In PZ2 (coinciding with the major lithological change between the two firsts units), pollen assemblages show a major vegetation change characterized by an abrupt drop of Ericaceae after 15.2 kyr followed by a decrease of *Myrica* and *Podocarpus* after 12 kyr. The montane vegetation, replaced by Elaeocarpaceae, *Macaranga*-*Mallotus*, Moraceae-Urticaceae undiff. and *Celtis,* shows a succession between the montane vegetation and the evergreen humid forest which reaches a maximum expansion after 11.8 kyr. The development of evergreen humid forest combined with a decrease of aquatic plants, suggest an important precipitation increase in comparison to PZ1. Although the evergreen humid forest remains stable until 0.9 kyr several distinct evergreen forest successions can be observed. For example, the evergreen humid forest from mid altitude is defined by the genus *Tambourissa*-*Weinmannia*[18] and our pollen assemblages illustrate maximum values of *Weinmannia* between 11.8 kyr and 5.5 kyr. Afterwards, several pioneer taxa, typical of forest edges or increased disturbances such as *Macaranga*-*Mallotus* or *Trema,* increase slightly[19,21].

From 5.5 kyr, contemporary to change in mean particle size (coarsening), an increase of aquatic plants is also recorded in particular by *Potamogeton,* reflecting again a lake with large shallow areas certainly related to a decrease of lake level and precipitation that also may explain the observed change in forest composition. In PZ3, starting from ~0.9 kyr, the evergreen humid forest shows an abrupt decrease at the expanse of Poaceae combined with an increase of Cyperaceae. Today, while the evergreen humid forest is still dominant in Montagne d'Ambre, this abrupt change may indicate the beginning of the colonization of the margins of the lake by sedges and peat bog which is currently growing on the lake.

Combined with pollen data, the charcoal record allows to reconstruct fire history in the environment surrounding our study site and around Montagne d'Ambre, improving our understanding of the regional environmental changes. A first increase of charcoal influx between 14.5 and 13.5 kyr is occurring at the beginning of the second pollen zone during the transition between the montane vegetation and the evergreen humid forest. This first episode might be related to the important climate change during this transition that may have produced fire prone vegetation (with increase of *Podocarpus* combined with *Myrica*), increase of thunderstorms with lightening frequency and/or the first development of suitable amounts of combustible biomass in the region. However, based on the relatively low values of charcoal influx, local fire occurrences at the catchment of the study lake can be excluded. After 13 kyr with the full development of evergreen humid forest, charcoal particles are almost absent until the late Holocene. Just few particles are counted during the late Holocene before a continuous occurrence starts simultaneously with the change to PZ3 at 0.9 kyr and charcoal shows a dominance of graminoid pieces. While the modern evergreen humid forest in Montagne d'Ambre is not prone to fires, the increased fire activity during the last millennium is most likely the result of a regional increase of fires in the lowland areas surrounding Montagne d'Ambre. This marked change is certainly related to a significant increase of human impact and activities in the region as evidenced by archeological data and other records from northwestern Madagascar[22–24]. In addition, the abrupt increase recorded by Poaceae is also certainly reflecting a regional signal characterized by an openness of the lowland landscape related to the fire activity which increased since 0.9 kyr.

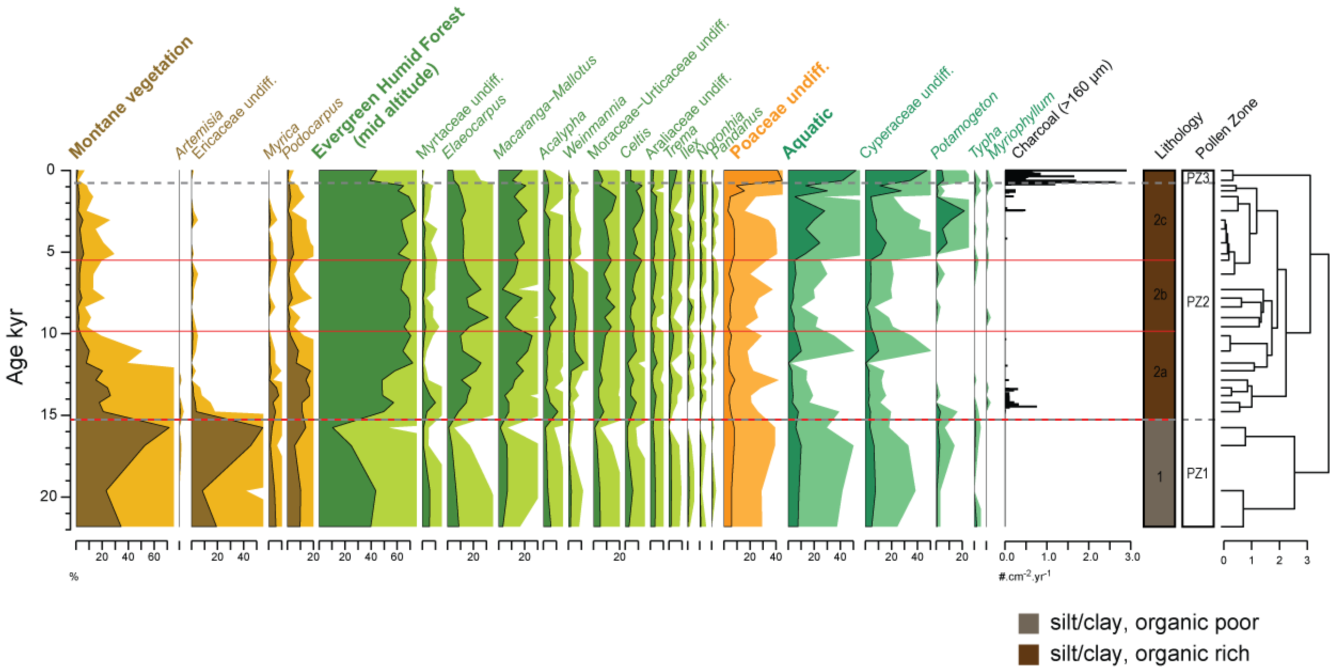

Fig. S4. Synthetic pollen diagram and influx of charcoal particles from core LM1B against the age scale indicated in calendar kiloyear before present (kyr). Vegetation groups are indicated in bold and correspond to the sum of the taxa plotted with the same color (light color curves correspond to dark colors curves multiplied by 5). The pollen record was subdivided into three significant pollen zones (PZ) by applying a constrained cluster analysis by sum of squares analysis performed on terrestrial pollen taxa[25] The grey dashed line and the white bar on the right side represent the PZ. The bar on the right side and the red lines represent the main sedimentological units and sub-units. The pollen diagram and cluster analyses were performed using the RStudio software (Version 1.2.1335) running the package "Rioja" (Version 0.9-21)[26].

2. Supplementary information for the demographic reconstructions

2.1. Supplementary Methods

2.1.1. Mouse lemurs capture. Given that little is known about the distribution and ecological preferences of *M. arnholdi*, two survey methods were initially used to verify its presence: nocturnal distance sampling and trapping, which were performed along 1 km length transects established at different locations close to the lakes (see [27] for details about the survey methods). A total of 586 Sherman Traps (Sherman Traps Inc, Tallahassee, FL, USA) baited with banana were installed overnight in Mahasarika during six nights, and 454 traps were installed in Fantany during five nights. However, this method was not successful, and all animals were subsequently captured by hand near those trees that they used during nighttime activity. The animals were kept in a Sherman Trap with a piece of banana and moved to the camping site where they were handled during the next morning. All the animals were released at dusk on the day of handling at the same place where they were captured. Small ear biopsies (approx. 2 – 3 mm^2) were taken from all captured animals for genomic analyses, following an individual marking pattern that secured individual identification in each study site. Tissue samples were stored in Queen's lysis buffer[28], were preserved at room temperature during the field season and subsequently at -20 °C in the laboratory.

2.1.2. RADseq library. DNA concentration was initially estimated with the Qubit® Fluorometer (Life Technologies), using the dsDNA HS (High Sensitivity) Assay Kit. RADseq libraries were prepared using 200 ng of genomic DNA and the TruSeqNano DNA HT kit (Illumina). The restriction enzyme Sbf I was used to generate the DNA fragments. Libraries were prepared in sets of 24 samples sorted based on their original DNA concentration. Each sample was ligated to one of 48 available P1 adapters with a unique 5 base pair (bp) molecular identifier (MID) for correct discrimination of the samples after sequencing. All fragments were randomly sheared using a Covaris M220 ultrasonicator, resulting in fragments with an average size of 550 bp. Sheared DNA fragments were ligated to the P2 adapter and all fragments with both P1 and P2 adapters were amplified in 10 Polymerase Chain Reaction (PCR) cycles. DNA concentration and fragments sizes of the amplified libraries were verified on a Qubit® Fluorometer and on a Bioanalyzer 2100, respectively. The libraries were sequenced using 150bp paired-end reads on an Illumina HiSeq3000 at the GeT-PlaGe platform (Toulouse, France) (for details about RADseq method see [29]).

2.1.3. Pipeline for the analyses of raw RADseq reads. Raw reads were initially demultiplexed by individual molecular identifiers with *splitbc* from the FASTX-toolkit (http://hannonlab.cshl.edu/fastx_toolkit/). The quality of the raw reads was checked with FastQC v0.11.7 (http://www.bioinformatics.bbsrc.ac.uk/projects/fastqc) and the raw data were filtered with Trimmomatic v0.36[30]. Illumina adapters were removed from the reads (ILLUMINACLIP:2:30:10)

and low quality bases on read ends were removed (LEADING:3 and TRAILING:3). A minimum 4-base sliding window trimming was performed to cut the bases with quality score below 15 (SLIDINGWINDOW:4:15). Reads with less than 60 bp length after the cleaning steps were removed from the analyses (MINLEN:60). BWA-MEM (http://bio-bwa.sourceforge.net/) was used to align the paired-end reads of each sample to a high-coverage genome assembly of *Microcebus murinus*[31] (genome coverage: 221.6X; GenBank Assembly accession number: GCA_000165445.3) and to a *M. murinus* mitogenome[32] (17114 bp; GenBank Assembly accession number: KR911908.1). Only the reads that mapped against the autosomal chromosomes were kept in the pipeline. In order to decrease the computational effort, the alignments were converted from the Sequence Alignment Map (SAM) format to the corresponding binary version (BAM) with SAMtools v1.8[33]. PCR duplicates created during PCR amplification were finally removed using SAMtools v1.8[33].

Next-Generation Sequencing platforms can generate large amounts of sequencing data but are prone to sequencing errors[34–36]. ANGSD (Analyzing Next Generation Sequencing Data)[36] provides an analytical framework that includes multiple methods using genotype likelihoods (i.e., marginal probability of the sequencing data given a genotype in a particular individual in a particular site) during downstream analyses[36]. Genotype likelihoods retain information about uncertainty in base calls, which enables to control some issues commonly associated with RADseq datasets (e.g. unevenness in sequencing depth and allele dropout)[34–36]. Therefore, ANGSD was used to call genotype likelihoods in all analyses performed with the RADseq dataset.

2.1.4. Whole-genome sequencing library, data filtering and command lines for *PSMC* and *IICR*. Libraries of isolated DNA from the two mouse lemurs were prepared using NEBNext Ultra DNA Library Prep Kit for Illumina (New England BioLabs, Ipswich, MA, USA). Samples were sheared by a focused-ultrasonicator (Covaris M220, Woburn, Massachusetts, USA) and underwent indexing and size selection according to the manufacturer's recommendations. Whole genome sequencing was performed on an Illumina NextSeq 500 (Illumina, San Diego, CA, USA) for 300 cycles in paired-end mode. Visual quality control of whole-genome sequencing data was performed using fastqc, version 0.11.7 (http://www.bioinformatics.babraham.ac.uk/projects/fastqc/). Reads were trimmed using PRINSEQ version 0.20.4[37] and mapped to the reference genome *M. murinus*[31] (genome coverage: 221.6X; GenBank Assembly accession number: GCA_000165445.3) using BWA version 0.7.17[38]. *PSMC* analyses were done using the following command line: -N30 –t5 –r5 –p "4+25*2+4+6" -D100 –d3 –q30.

2.1.5. Relatedness analyses. Two alleles are identical by descent if they recently descended from a common ancestral allele[39,40]. The probability of two individuals sharing 0, 1 or 2 alleles from a single ancestor at any locus is expressed by the identity-by-descent (IBD) coefficients k_0, k_1 and k_2, respectively[39]. The software NGSrelate[40] allows to estimate those IBD coefficients based on the

allele frequencies per site. Allele frequencies were calculated using ANGSD[36] by inferring major and minor alleles and considering only sites with a p-value < $1e^{-6}$ and a minor allele frequency > 0.05. The IBD coefficients among individuals were calculated with NGSrelate. The relatedness categories between individuals (i.e. 1°, 2° and 3° degree of relatedness or unrelated) were inferred based on the IBD coefficients that were compared to the expected IBD probabilities (Parent-offspring: $k_0 = 0$, $k_1 = 1$ and $k_2 = 0$; Full sibs: $k_0 = 0.25$, $k_1 = 0.50$ and $k_2 = 0.25$)[39].

2.1.6. Demographic methods assumptions. The *Stairway Plot*[41] is a widely use tool that makes use of the Site Frequency Spectrum generated from population genomic data to infer population size changes. It estimates a series of population mutation rates ($\theta = 4N_e\mu$), following a multi-epoch demographic model, where epochs coincide with coalescent events[41]. Changes in N_e through time are estimated based on the estimation of θ. Consequently, the plot is not continuous, but describes discrete blocks of times (epochs), where the number of the epochs is dependent of the number of possible coalescent events in the dataset (i.e. the number of individuals and the number of SNPs)[42]. Alternatively, the *PSMC*[43] makes use of the whole genome of a single diploid individual to infer population size changes, and it relies on the coalescent theory to estimate the time to the most recent common ancestor (TMRCA) of two alleles at a given locus[43]. It has been stated that this method is more informative about events occurring in the distant past[41,43–47]. Both the *Stairway Plot* and the *PSMC* assume that the genomic data used for the analyses come from a panmictic population. Simulating the *Inverse Instantaneous Coalescence Rate* (*IICR*)[48] under various parameters allows to investigate the effect of population structure and therefore deviations from panmictic conditions on demographic inferences inferred by the *PSMC* method. The *IICR* function is equivalent to population size changes for a genomic dataset from an unstructured population. Given structured populations, the *IIRC* varies in response to changes in connectivity (gene flow) between demes, to the number of islands in an n-island model of migration and also to the number of connectivity changes[48,49]. Finally, the *fastsimcoal2*[50] is a composite-likelihood method that allows to compare alternative evolutionary scenarios from a Joint Site Frequency Spectrum (e.g. 2d-SFS or 3d-SFS), where the number of dimensions refers to the number of populations compared in the models[51]. The SFS-based methods require less sequence data per individual but a greater number of individuals to estimate a well derived SFS. A minimum of 10 individuals per population is typically used and was achieved in our study[45,50,52].

2.1.7. *fastsimcoal2* command options. We ran *fastsimcoal2* v.2.6[50] using 200,000 coalescent simulations per sets of parameters (-n 200 000), 40 ECM cycles during parameter estimation from the SFS (-L 40) and –C 1 as the threshold for the observed SFS entry count. The additional –M and –m options were used to perform parameter estimation from the folded 2d-SFS with the maximum composite likelihood. Details of model design and model selection are described under

section 2.2.3. With the exception of the migration rate, wide search ranges with uniform distributions were used for all models (Table S4). A total of 100 independent *fastsimcoal2* runs were performed for all 13 demographic models to determine the parameter estimates that maximize the composite-likelihood[50]. As a last step, *fastsimcoal2* was used to generate 20 bootstrap SFS for the three best ranked demographic models, considering the parameters that maximized the likelihood for each respective model. A total of 20 independent *fastsimcoal2* runs were performed *a posteriori* with each of the 20 generated bootstrap SFS for each model, using the previous command options (-L 40 -n 200000 -m -M -C1). The parameter estimates with the highest likelihood from each independent run were lastly used to calculate the 95% confidence intervals.

2.1.8. Linkage Disequilibrium. The use of linked sites in the *fastsimcoal2* approach does not bias parameter estimation, since composite-likelihoods converge to the correct parameters that maximize the likelihood, but it may bias the likelihood estimation[50,52]. In order to account for this potential source of bias, we inspected the entire SNP distribution across the *M. arnholdi* genome, and we thereby confirmed that the RADSeq related SNPs were spread out widely and occurred across all 32 autosomal chromosomes (See Fig. S5). Therefore, we kept all SNPs of our dataset for the *fastsimcoal2* analyses, but we are aware that the estimated AIC values should be interpreted with caution. Consequently, instead of considering only the best-ranked model for *M. arnholdi*, we compared the fit of the three best ranked demographic models (see section 2.2.3.).

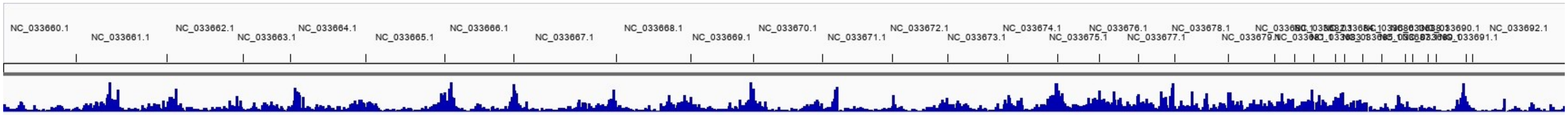

Fig. S5. **SNP distribution across the entire *M. arnholdi* genome**. The figure shows that SNPs were widely scattered across the 32 autosomal chromosomes.

2.1.9. General considerations about simulating the *IICR*. The figure below (Fig. S6) represents the *IICR* under the structure model that best fitted the *IICR* inferred by the *PSMC* (Figure 3b) for Fantany:

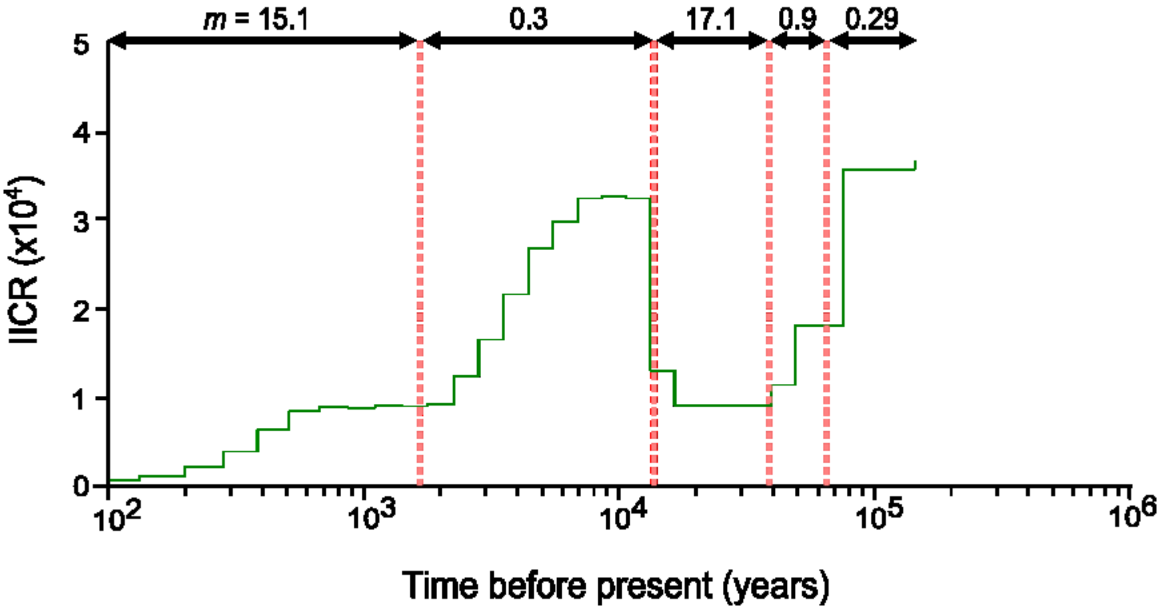

Fig. S6. ***IICR*-simulation under the structure model for Fantany.** The figure represents the *IICR* under an n-island model of migration with a constant population size that best fitted the *IICR* inferred by the *PSMC*. Vertical red lines mark the times of change in population connectivity. The arrows on the top bar represents the relative levels of migration rate inferred for each time period.

The present curve was computed with the python script available at: https://github.com/willyrv/IICREstimator[53] considering the following ms command:

ms 2 1000000 -T -L -I 29 2 0 0 0 0 0 0 0 0 0 0 0 0 0 0 0 0 0 0 0 0 0 0 0 0 0 0 0 15.0928 -eN 0 1.0 -eM 0.6048 0.3413 -eN 0.6048 1.0 -eM 4.542 17.1249 -eN 4.542 1.0 -eM 13.682 0.9428 -eN 13.682 1.0 -eM 22.2268 0.2924 -eN 22.2268 1.0

This ms command specifies that Fantany was constant at size N_0 thought the time, but underwent four historical changes in population connectivity. The demographic parameters are specified by the following command options:

–I (number of islands) = 29

–eN *t x* (set all subpopulations to size $x * N_0$), where x = 1.0 thought the time

–eM t *m* (set all elements of the migration matrix to *m*/(npop−1) at time t), where *m* = 15.0928; 0.3413; 17.1249; 0.9428; 0.2924 (backwards in time)
t (specifies the time of a given demographic event in units of $4N_0$ generations), where *t* = 0; 0.6048; 4.542; 13.682; 22.2268 (backwards in time)

For a generation time of 2.5 years and an N_0 of ~ 292, the changes in population connectivity took place: ~1.8, 13, 40 and 65 kyr before present (marked by vertical lines in the Fig. S6).

Altogether, the present *IICR* simulations suggest higher levels of population connectivity before the AHP (between 40 – 13 kyr; *m* = 17.1249), and a decrease of population connectivity during the AHP and beyond (between 13 –1.8 kyr; *m* = 0.3413).

For additional information about the hand-fitting *IICR* please see:
https://github.com/willyrv/IICREstimator/blob/master/tutorial_handfitting_IICR.md

2.2. Supplementary Results

2.2.1. Genomic datasets. Of the 46 samples (14 from Mahasarika and 32 from Fantany) sent for RADseq sequencing, a total of 38 samples (12 individuals from Mahasarika and 26 from Fantany) passed all quality filters, i.e., exhibited the mean sequencing coverage required for the analyses with the ANGSD framework tools (> 4X; see[36,40,54] and were unrelated to other individuals of our dataset (Table S2). An average of 10,008,635 ± 3,374,440 (SD; standard deviation) Illumina reads per individual were obtained for the final RADseq dataset (n = 38). After applying the quality filters (e.g. removing Illumina barcodes, discarding low quality reads and trimming), an average of 8,692,396 ± 2,920,414 reads per individual was retained in the analyses. Of these, an average of 7,590,190 ± 2,589,226 reads per individual was successfully mapped to the *M. murinus* reference genome. After PCR duplicate removal, an average of 5,940,281 ± 2,050,423 reads per individual (Table S3) were retained for the further analyses. After all cleaning steps, the maximum number of sites used for the subsequent analyses were 58,219,228 sites (490,778 variant sites) for the larger dataset (*Stairway Plot*; n = 38) and 62,316,703 sites (737,670 variant sites) for the smallest dataset (*fastsimcoal2*; n = 20).

Whole-genome sequencing and mapping of the two *M. arnholdi* individuals resulted in 37,016,904,234 (Mahasarika) and 43,604,237,434 (Fantany) bases mapped as well as an error rate of 3.90 X 10^{-2} (Mahasarika) and 3.96 X 10^{-2} (Fantany). The mean depth of coverage ranged between 13.72X (Mahasarika) and 16.01X (Fantany). For *PSMC* analysis, a total of 2,138,200,000 and 2,146,600,000 sites were processed for Mahasarika and Fantany, respectively. For details about the list of individuals used for each demographic method see Table S2. All RADseq sequences obtained in this study are publicly available at Sequence Read Archive (NCBI) in the BioProject PRJNA560399 (Number accession: SAMN14854044 – SAMN14854081). Whole Genome sequences are available in the BioProject PRJNA632451 (Biosample: SAMN14909740 for Mahasarika and SAMN14909741 for Fantany).

2.2.2. Genetic structure in *M. arnholdi*. The analysis with NGSadmix[35] confirmed the existence of genetic structure among the two sites despite their relative proximity (18 km) and the continuous forest connecting them. Under the best partition of K = 2, all 12 individuals captured in Mahasarika were assigned to one of two clusters (blue) with an average membership (Q) coefficient of 100%, whereas all 26 individuals sampled in Fantany were grouped in the second cluster (red) with a Q coefficient ranging between 76-100% (Fig. 1b). At K = 3, the individuals from Mahasarika remained homogenous in one cluster, whereas all individuals from Fantany were assigned in varying proportions to the two other clusters (Fig. S7a). The occurrence of small-scale genetic structure has been observed in other mouse lemur species (e.g., in *M. murinus*[55] and *M. ravelobensis*[56,57] and may be an effect of small dispersal distances described for mouse lemur species (e.g., below 1 km for *M. murinus*)[55].

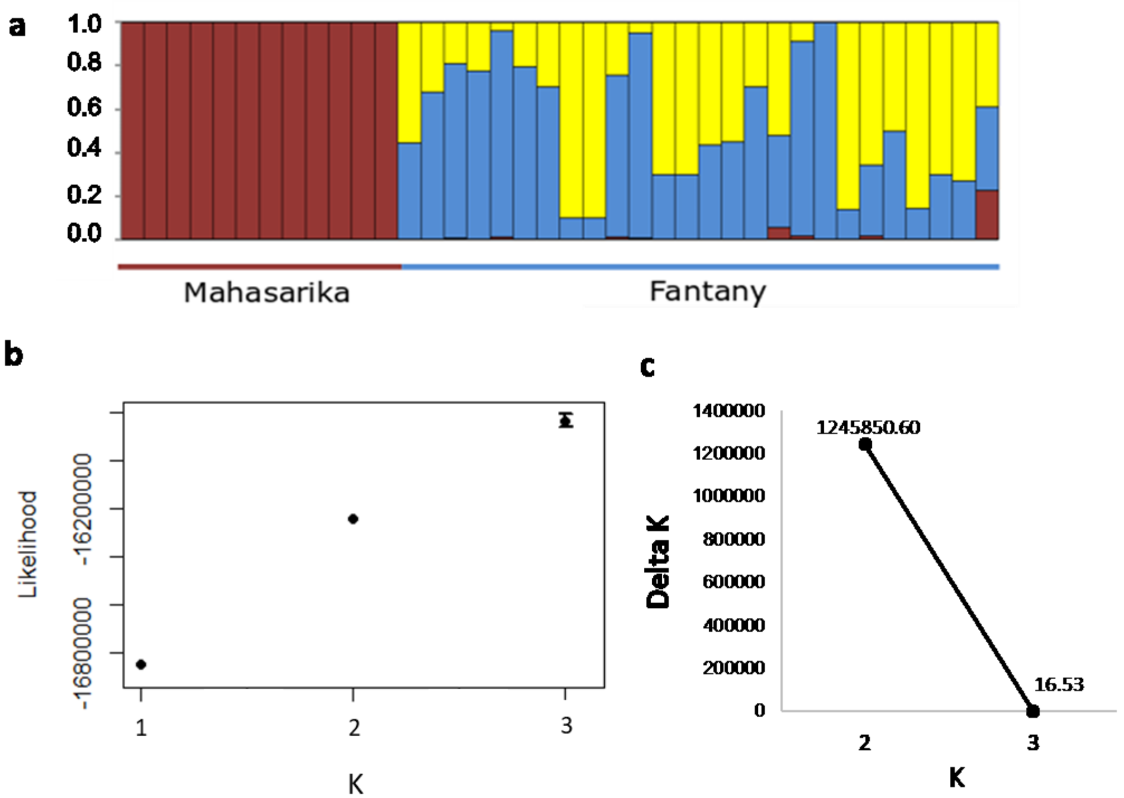

Fig. S7. **Number of clusters inferred by NGSadmix**. **a,** Clustering assignment of 38 individuals to three genetic clusters (K = 3) using genotype likelihoods. Each individual is represented by a single vertical bar and each color represents a distinct genetic cluster. Samples are sorted according to sampling site. **b,** Likelihood results under K = 1 - 3. **c,** Delta K estimation following the method of Evanno[58] over 10 replicate NGSadmix runs for each K value. The best K value for *M. arnholdi* is K = 2.

2.2.3. Model selection with *fastsimcoal2.* The demographic history of *M. arnholdi* was first explored with five simple demographic models, assuming a panmictic population with constant population size – *null model* (M1); a panmictic population with a single population size change – *one size change model* (M2); a panmictic population that recently split in several demes – *recently structured model* (M3); an ancient structured population – *ancient structured model* (M4); and an ancient structured population that underwent changes in connectivity through time – *change in connectivity model* (M5; see Fig. S8). The lowest ΔAIC values were observed for the *change in connectivity model* (M5) and the *recently structured model* (M3), while the two models assuming panmixia displayed the highest AIC and likelihood values (see Table S6). The two structured models had similar ΔAIC, and the parameter estimates for both models point towards the same demographic scenario, since the migration rates were relatively high for the *ancient structured model* (see Table S5). The two models M3 and M5 were therefore chosen as basis for defining more complex models in the next step.

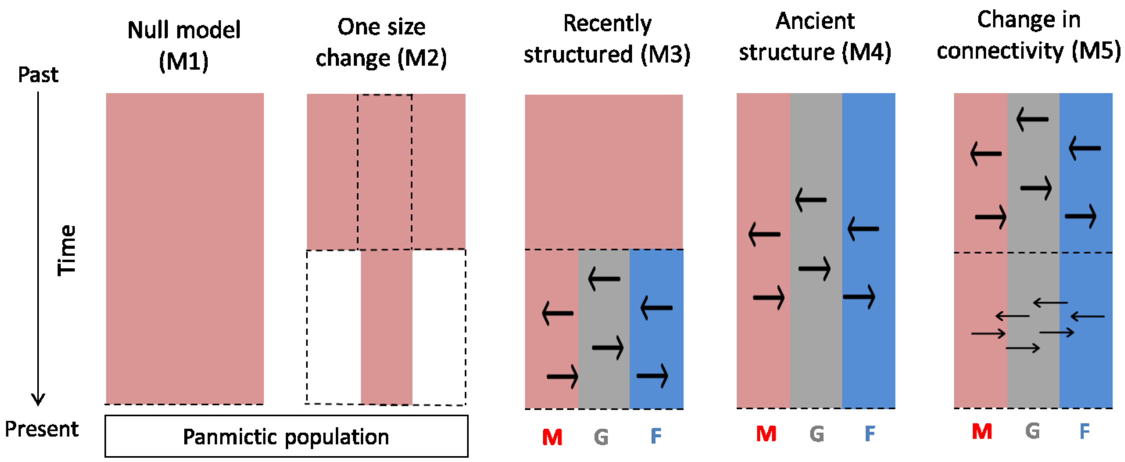

Fig. S8. Illustration of five simple demographic models compared with *fastsimcoal2* approach (step 1). The simplest scenario assumes the existence of a panmictic population without size change (null model, M1) or with a single size change (one size change model, M2). The third scenario relies on the existence of a panmictic population that recently split in several demes (recently structured model, M3). The remaining two scenarios assume the existence of an ancient structured population without changes in connectivity (ancient structured model, M4) or with changes in connectivity through time (change model, M5). The lowest ΔAIC values were observed for M3 and M5 (see Table S6).

As a next step, four demographic models assuming recently structured populations (M6 – M9, Fig. S9a) and four models incorporating changes in connectivity (M10 – M13, Fig. S9b) with additional changes in population size were compared. Within the "*recently structured models*" category, the following scenarios were tested: occurrence of a single recent population bottleneck (M6); two consecutive population bottlenecks (M7); or an asymmetric population bottleneck (M8)

after the populations become structured. M8 assumes that only the population sampled at the forest ecotone (Fantany) underwent a first reduction in size that was followed by a recent decline of all populations. Finally, we tested for the occurrence of an ancient population expansion before the population become structured and declined (M9). Within the "*change in connectivity models"* category, the following scenarios were tested: occurrence of a population bottleneck accompanied by a change in migration rate (M11); a population bottleneck followed by one change in migration rate (M10); two successive bottlenecks accompanied by two changes in migration rate (M12); and an ancient population expansion followed by one subsequent change in migration rate (M13; see Fig. S9).

The comparison of the results for all 13 demographic models (Table S6) shows that: i) models assuming demographic changes (either population size or connectivity change) had a better fit than the null model (i.e. constant population size; M1); ii) all structured models (M3 – M13) had a better fit than entirely panmictic models (M1 and M2), supporting the existence of population genetic structure; iii) recently structured models with population size change (M6 – M9) had a better fit than structured models with both population size and connectivity changes (M10 – M13); iv) the three best ranked models (M6 - M8) suggested the existence of a large ancient population that became structured only recently and underwent one or two declines in population size.

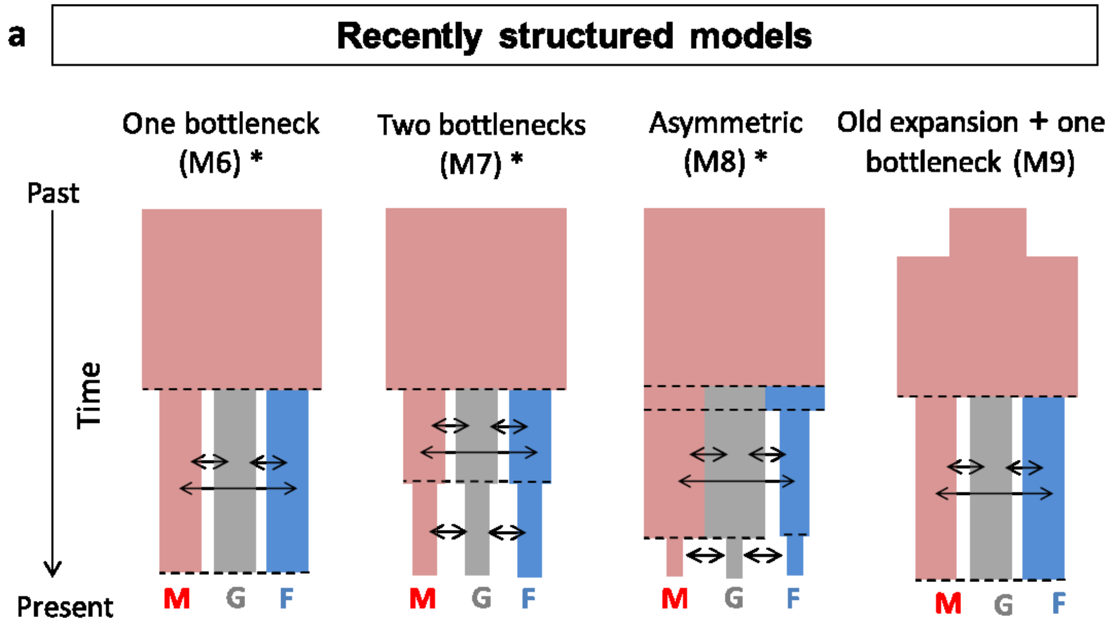

* Best ranked demographic models

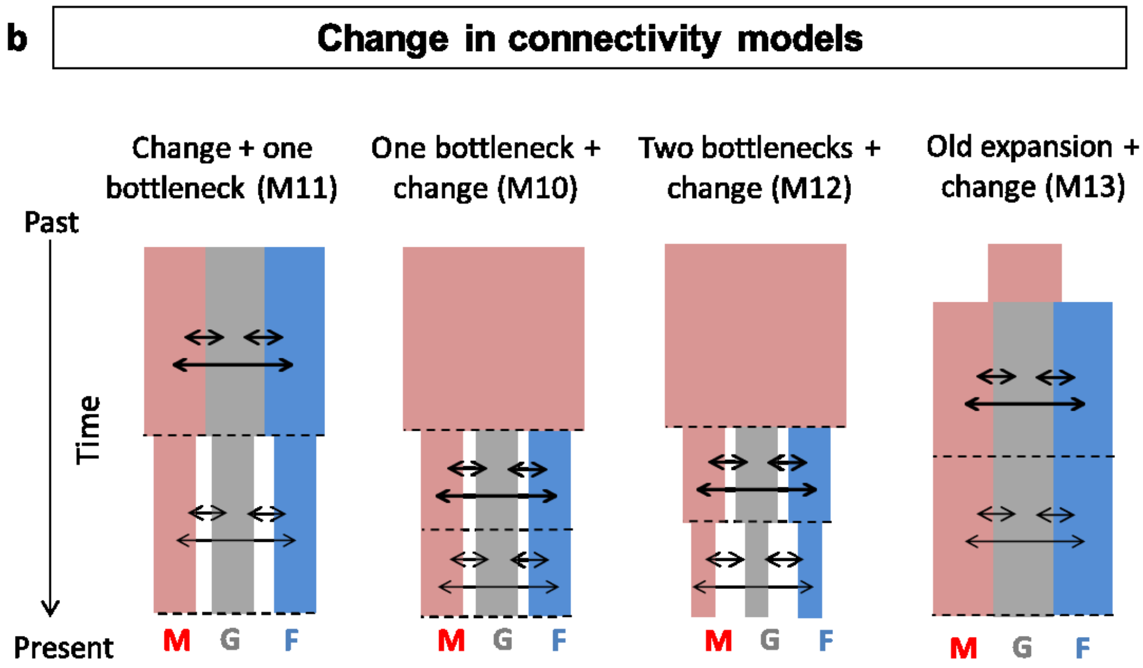

Fig. S9. Illustration of the eight more complex demographic scenarios compared with *fastsimcoal*2 approach (step 2). These models are divided into the recently structured and change in connectivity model categories. **a,** The *"recently structured model"* category includes four scenarios with a single recent population bottleneck (M6), two population bottlenecks (M7), an asymmetric population bottleneck (M8), or an old population expansion combined with one recent bottleneck (M9). **b,** The "*change in connectivity models"* compared four scenarios that assumed the occurrence of a population bottleneck accompanied by a change in connectivity (M11), one population bottleneck followed by one change in connectivity (M10), two bottlenecks accompanied by one change in connectivity (M12), and an ancient population expansion followed by one change in connectivity (M13). Overall, the models with the lowest ΔAIC values were models M6, M7 and M8 from the "*recently structured model*" category.

The three best ranked models (M6 – M8; Fig. S10) exhibited similar ΔAIC values and a good fit between the expected and observed Site Frequency Spectrum (marginal 1d-SFS and 2d-SFS; Fig. S11 – S13). Consequently, all three models were kept for parameter estimations (Table 1, Table S7). Of these, only the model assuming the occurrence of two successive population bottlenecks (M7; Table 1) was kept as the best one, because the maximum likelihood parameter estimates for the other two models (M6 and M8; Table S7) fell all outside the 95% confidence intervals generated by block-bootstrap.

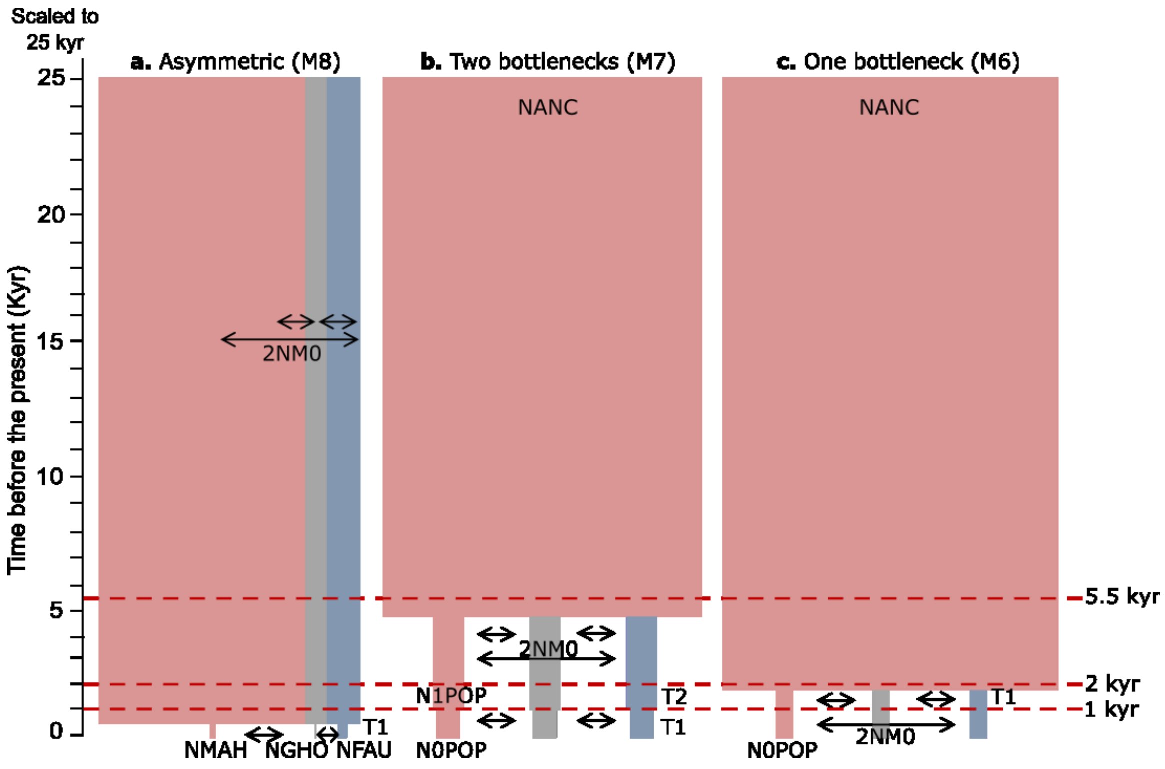

Fig. S10. Schematic illustration of the three demographic models with the lowest AIC (*fastsimcoal2*). For direct comparison of the inferred demographic history of *M. arnholdi* with the available paleoecological data, the models were illustrated only across the last 25 kyr. **a,** M8 infers the occurrence of one asymmetric population bottleneck; **b,** M7 suggests the occurrence of two consecutive population bottlenecks; **c,** M6 supports the occurrence of a single population bottleneck. The different populations are represented by distinct colors. An additional population ("Ghost population", grey) was included to represent *M. arnholdi* not covered by our sampling scheme. The rectangle width is proportional to the estimated N_e. The occurrence of gene flow is indicated by arrows. N0POP = effective population size for each population at present time; N1POP = effective population size for each population before a given demographic event; NANC = ancestral population size; NMAH, NFAU and NGHO = effective population size of populations Mahasarika, Fantany and Ghost, respectively. 2NM0 = average number of haploid immigrants entering the population per generation. T = time of a given demographic event, where T1 corresponds to the most recent time period and T2 to the older time period. Parameter estimates are given in the Tables 1 (M7) and S7 (M6 and M8).

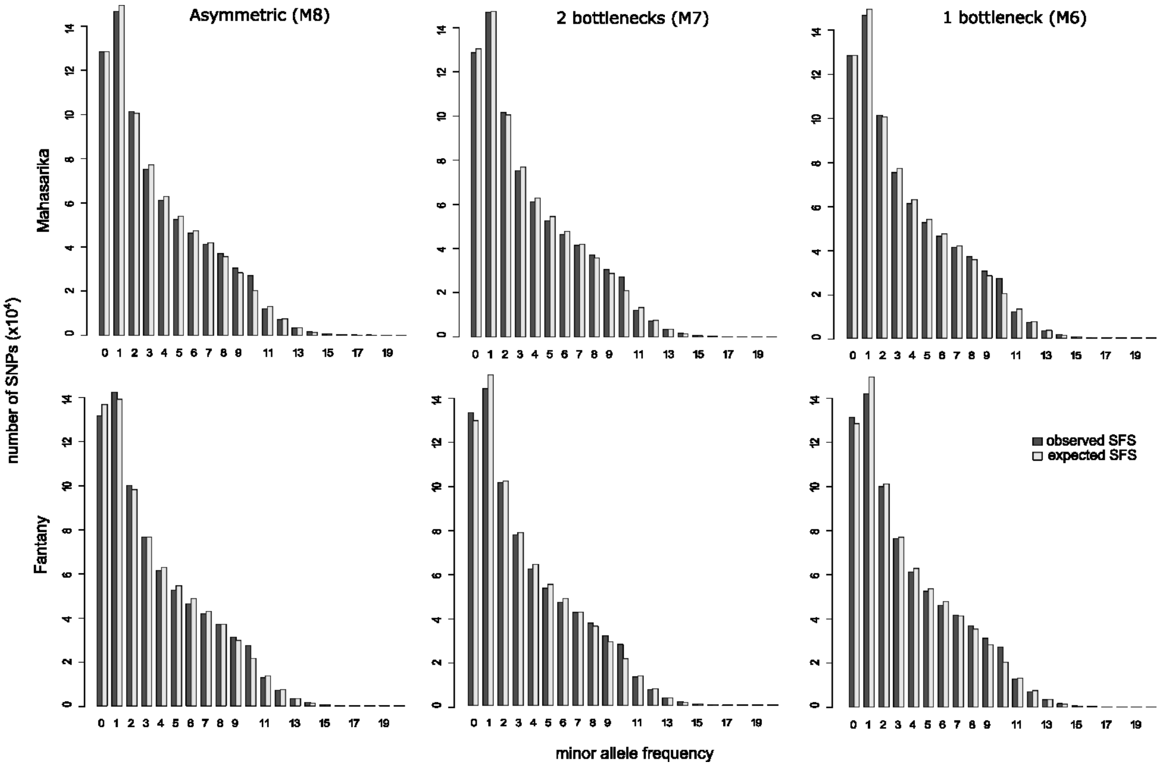

Fig. S11. **Fit of the expected marginal 1d-SFS under the three best ranked demographic models with *fastsimcoal2* for Mahasarika (upper panel) and Fantany (lower panel)**. The marginal SFS is obtained by summing all the entries of the 2d-SFS with a given frequency in one population and discarding the monomorphic sites across all samples. All demographic models exhibited a good fit with the observed SFS.

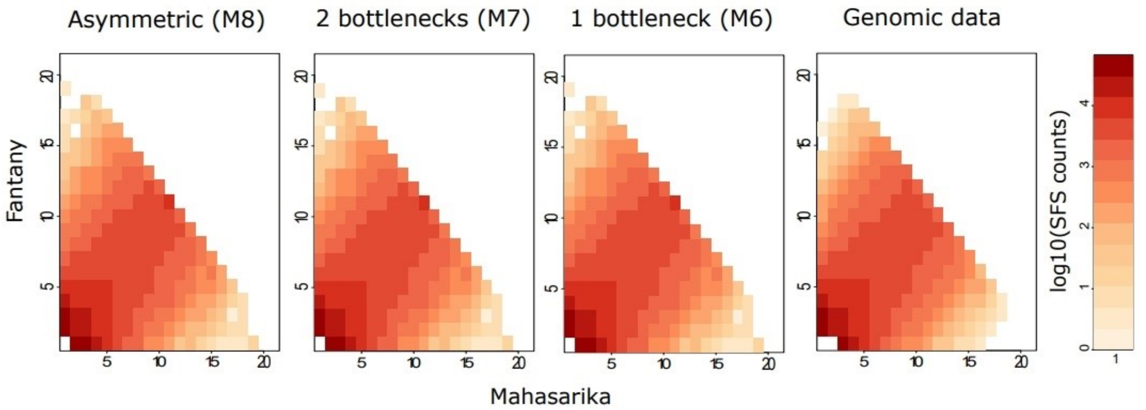

Fig. S12. **Comparison of the expected pairwise 2d-SFS obtained for the three best ranked demographic models with *fastsimcoal2* and the observed 2d-SFS between both populations.** Each row shows the observed or expected 2d-SFS in log10 scale. The monomorphic sites across all samples were discarded. The expected SFS was multiplied by the total number of SNPs to be in the same scale as the observed 2d-SFS. The figure shows a good fit between the three demographic models and the genomic data.

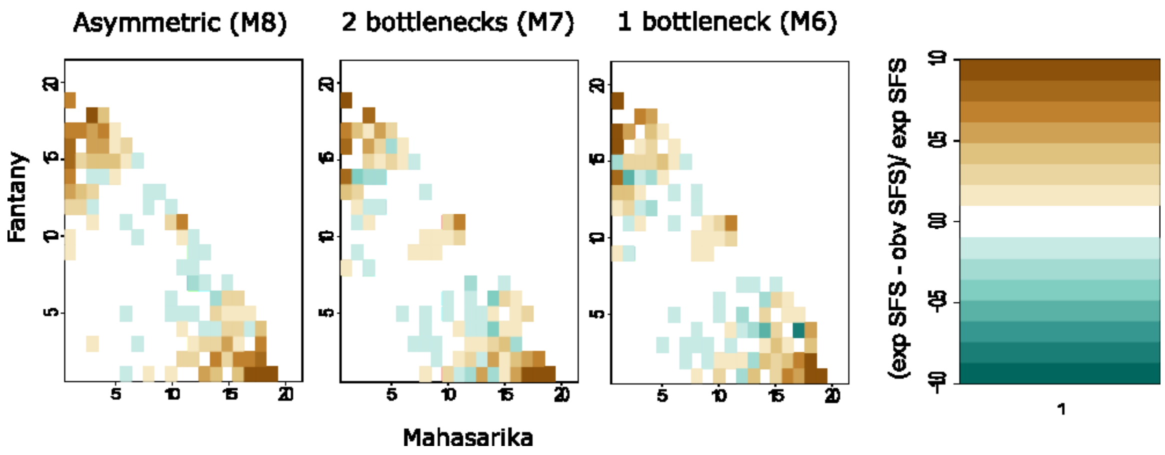

Fig. S13. Relative differences between the observed and expected SFS for the three best ranked demographic models with *fastsimcoal2*. Each row shows the relative differences between the observed and expected SFS. The figure show that all three demographic models are fitting the real data well.

2.2.4. Comparison of the alternative generation time estimates proposed for mouse lemurs. For all demographic methods, varying the generation time (1.0 year vs. 2.5 years vs. 4.5 years) strongly affected the chronology of demographic events. When varying the generation time within the *Stairway Plot* (Fig. S14), GT = 2.5 and 4.5 suggest a stable and large population size across the LGM which disagrees with the available knowledge on late Pleistocene vegetation dynamics. Even under GT = 1.0, the plots already suggested a population expansion during the LGM period, which reached its maximum population size already before or at the onset of the AHP (~ 14 kyr). Thereafter, the population sizes remained stable until the Mid-Holocene (~ 6 kyr). This period was followed by a massive population decline until the present days. In conclusion, GT = 1.0 and GT = 2.5 explained the recent population size dynamics of *M. arnholdi* relatively well, but none of the three GT values provided reliable datings for the older demographic events.

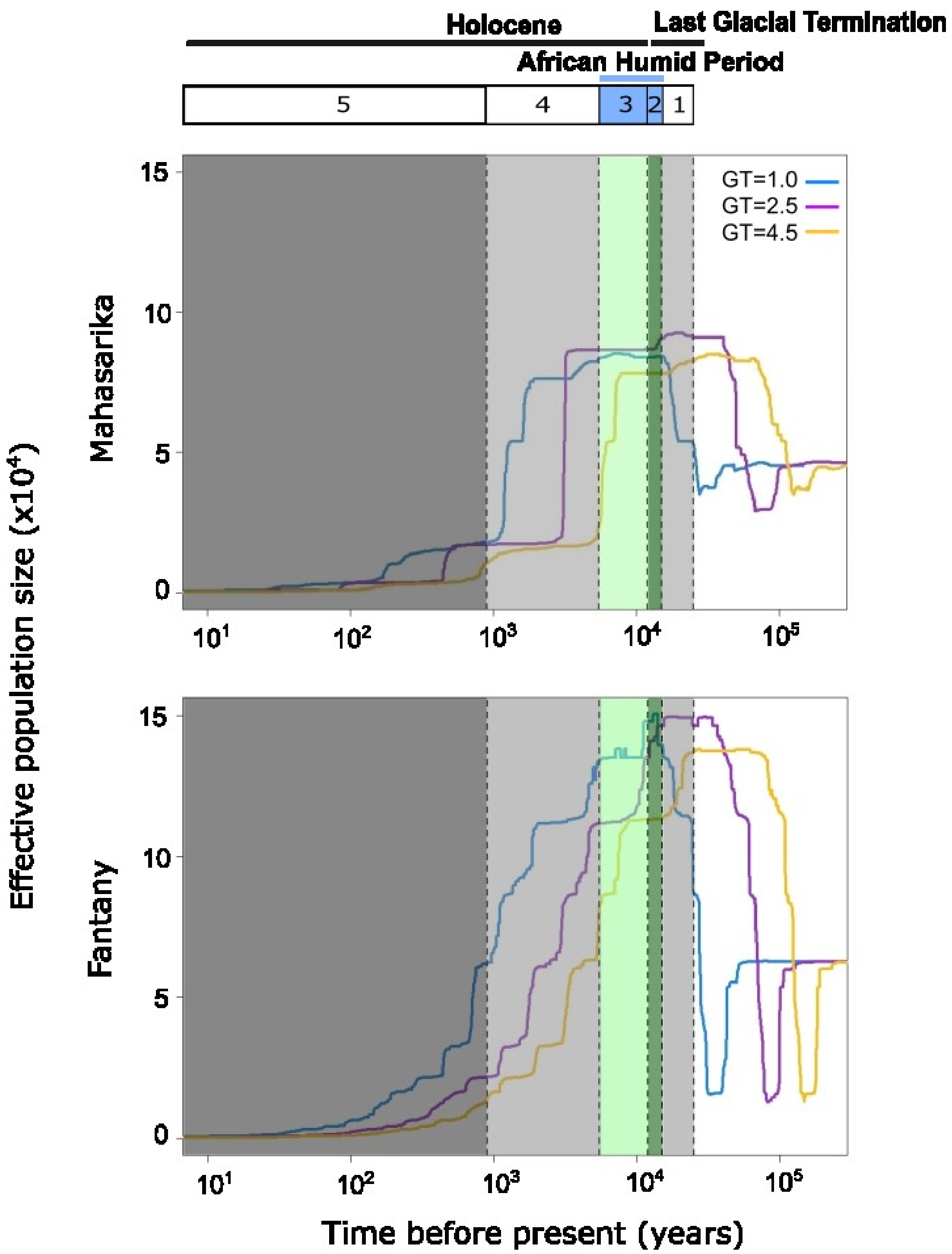

Fig. S14. Comparison of the demographic dynamics inferred for *M. arnholdi* with the *Stairway Plot* method, considering three generation times: 1.0 (blue), 2.5 (purple) and 4.5 years (yellow). The results for the different generation times are shown together with the five periods of vegetation and climatic changes revealed by the paleoecological analyses (in different color shadings). The legend on the top identifies the main periods of vegetation change derived from the paleoecological analyses (see main text).

When varying the generation time within *PSMC* (Fig. S15), under GT = 1.0, the oldest population bottleneck fell within the AHP which is unlikely. Considering GT = 4.5, the most recent population decline started during this period (~ 9 or 12 kyr). Both scenarios are not realistic, since the paleoenvironmental data show that the AHP was dominated by first expanding and then stable evergreen humid forest which are the preferred habitat for *M. arnholdi* at present times. In contrast, the results under GT = 2.5 fitted the older and younger vegetation dynamics best with an increase of N_e during the AHP followed by a population decline starting during the Mid-Holocene (Fantany ~ 7 kyr; Mahasarika ~ 5 kyr).

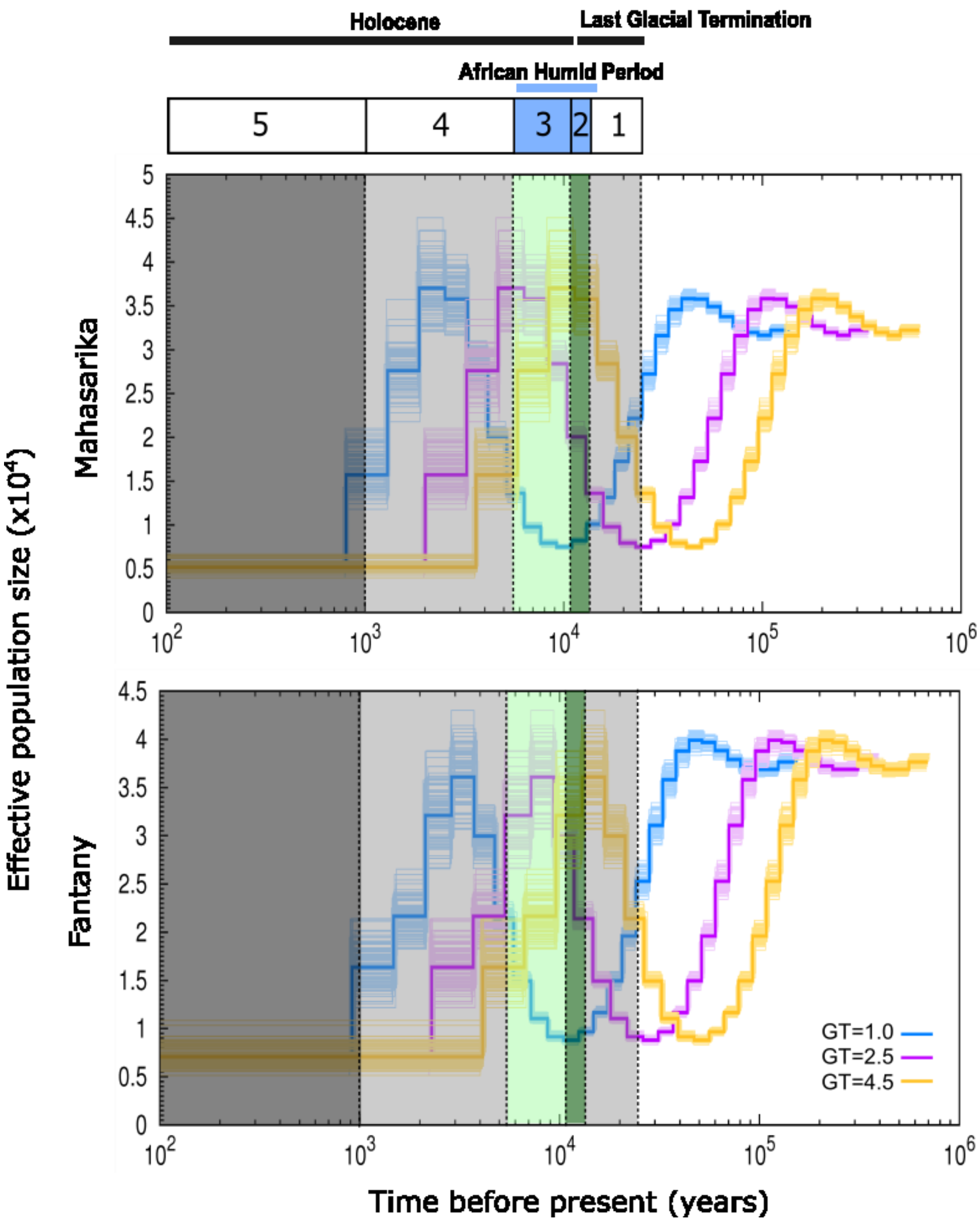

Fig. S15. **Comparison of the demographic dynamics inferred for *M. arnholdi* with the *PSMC* method, considering three generation times: 1.0 (blue), 2.5 (purple) and 4.5 years (yellow)**. The results for the different generation times are shown together with the five periods of vegetation and climatic changes revealed by the paleoecological analyses (different color shadings). The legend on the top identifies the main periods of vegetation changes derived from the paleoecological analyses (see main text).

When varying the generation time within the *IICR* under the structure model (Fig. S16), the GT = 1.0 suggest moderate levels of population connectivity during the LGM (26 – 16 kyr), increasing levels of connectivity during the AHP (16 – 5.3 kyr) and a decrease on connectivity after

the AHP termination (5.3 – 0.7 kyr; Fig. S16a). Considering GT = 2.5, our simulations points towards a period of higher connectivity starting before the LGM (~ 40 kyr) and lasting until the onset of the AHP (~13 kyr), which is followed by a period of reduced connectivity until recent times (13 – 1.8 kyr; Fig. S16b). These results are unlikely since the cooler conditions during the LGM would likely have resulted in lower connectivity, while the expansion of the evergreen humid forest during the AHP would have resulted in higher connectivity levels. For GT = 4.5, our simulations propose lower connectivity between the LGM until the late-Holocene (24 – 3.2 kyr; Fig. S16c). These results are also unrealistic since they would imply that population connectivity in *M. arnholdi* was not affected by the major vegetation changes revealed by the paleoenvironmental data at the onset (~ 15.2 kyr) and at the termination (~ 5.5 kyr) of the AHP.

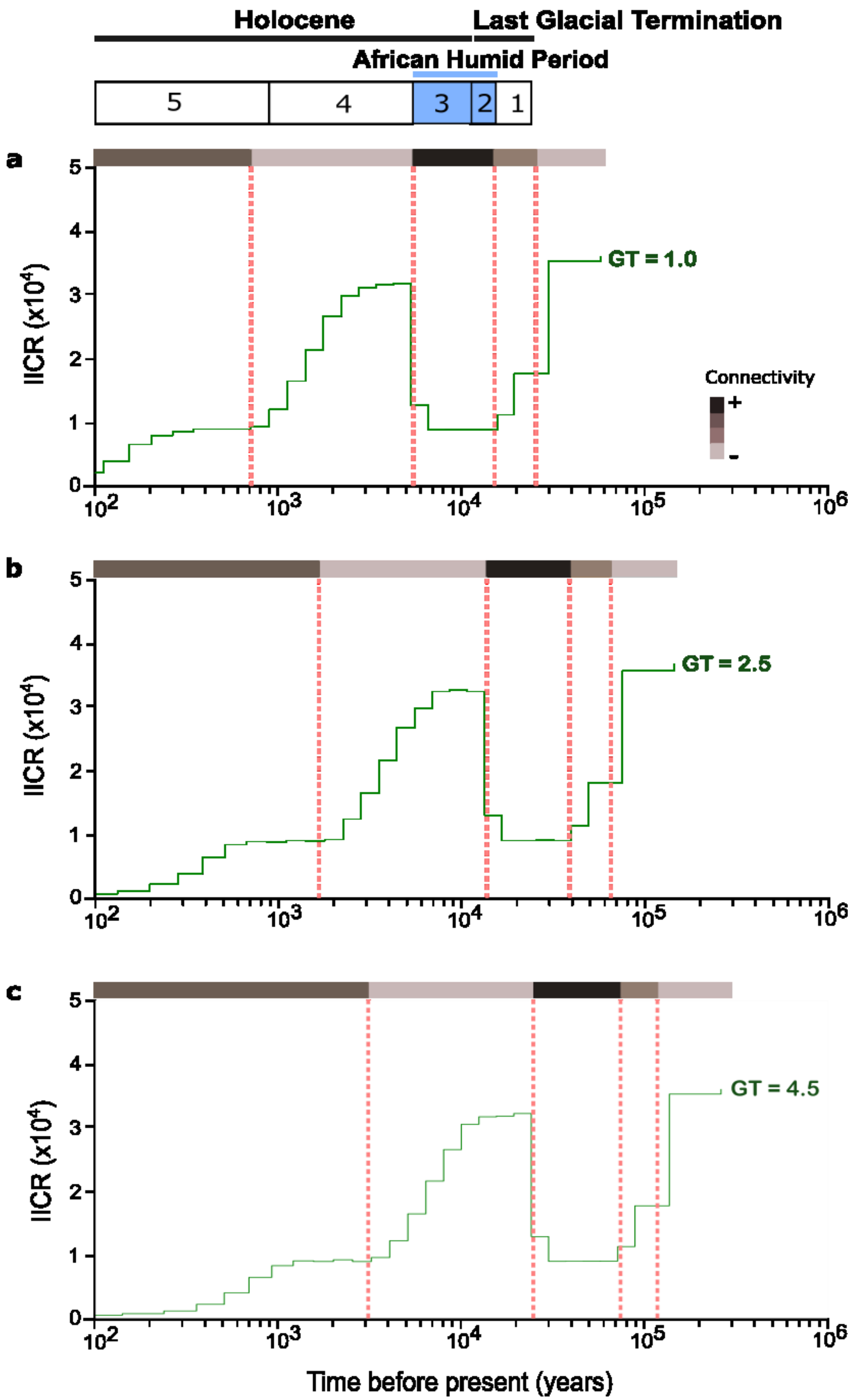

Fig. S16. Comparison of the *IICR* simulations under the structure model for Fantany considering three generation times. The effects of different generation times was compared to

the vegetation and climatic changes revealed by the paleoecological analyses. Vertical lines mark the times of change in connectivity. Horizontal bars on top in grey shades indicate relative levels of connectivity. **a,** Considering GT = 1.0, population connectivity in Fantany changed at 26, 16, 53 and 0.7 kyr. **b,** Considering GT = 2.5, population connectivity in Fantany changed at 65; 40; 13 and 1.8 kyr. **c,** Considering GT = 4.5, population connectivity in Fantany changed at 117, 72, 24 and 3.2 kyr.

When varying the generation time within *fastsimcoal2* for the best model (M7), a GT = 1.0 or 2.5 years would generate signals of mid- to late- Holocene population declines (Fig. S17). Whereas under GT = 1.0 the two consecutive population declines would have occurred during the last 2 kyr, GT = 2.5 corresponds to an earlier population decline starting at ~5 kyr and a recent decline starting at ~1 kyr. This GT = 2.5 scenario fits best to the paleoenvironmental reconstructions, as the earlier population bottleneck occurred at the termination of the AHP, and the most recent population bottleneck matches with the period of increase of fire activity. Under GT = 4.5 the earliest population decline would have started already during the AHP (~ 9 kyr) which is unlikely given the wide extend of evergreen humid forest at that time, while the most recent decline one would have fallen into the early Holocene (< 2 kyr).

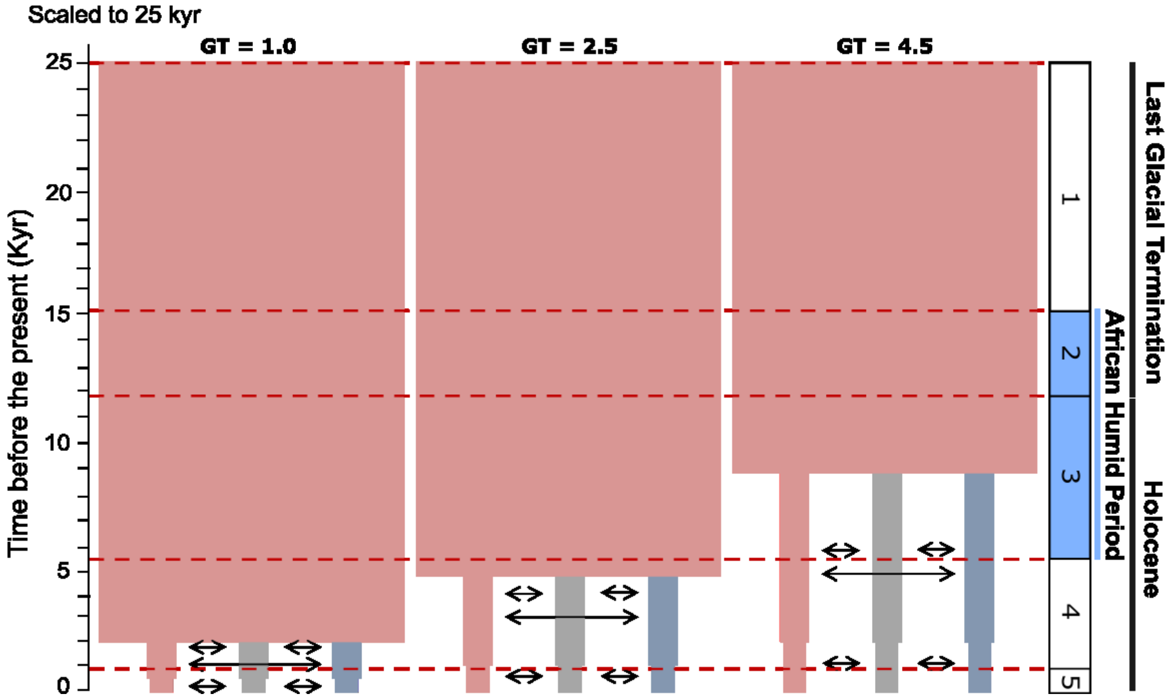

Fig. S17. Comparison of the timing of the demography events estimated for the best demographic model (M7) with *fastsimcoal2*, considering three generation times (GT = 1.0, 2.5, and 4.5 years). The effects of different generation times was compared to the vegetation and climatic changes revealed by the paleoecological analyses (right panel). For direct comparison of

the inferred demographic dynamics of *M. arnholdi* with the paleoecological data, the model was evaluated in more depth only across the last 25 kyr.

2.2.5. Impact of the minimum read depth in the *PSMC* analyses. The *PSMC* runs considering different minimum read depth options (–d1 to –d9) resulted in similar demographic curves for both study sites (Fig. S18). When decreasing the –d option from nine to one, the genome-wide coverage only decreased about 1X for both whole-genome sequences. These results suggests that the sites considered in the *PSMC* analyses were of good quality. Also, despite the mean genome-wide coverage differences (13.72X for Mahasarika and 16.01X for Fantany), the two sequences converged to an identical demographic history. Altogether, our results suggest that the *PSMC* inferences for *M. arnholdi* are likely not strongly biased by the relatively low mean genome-wide coverage.

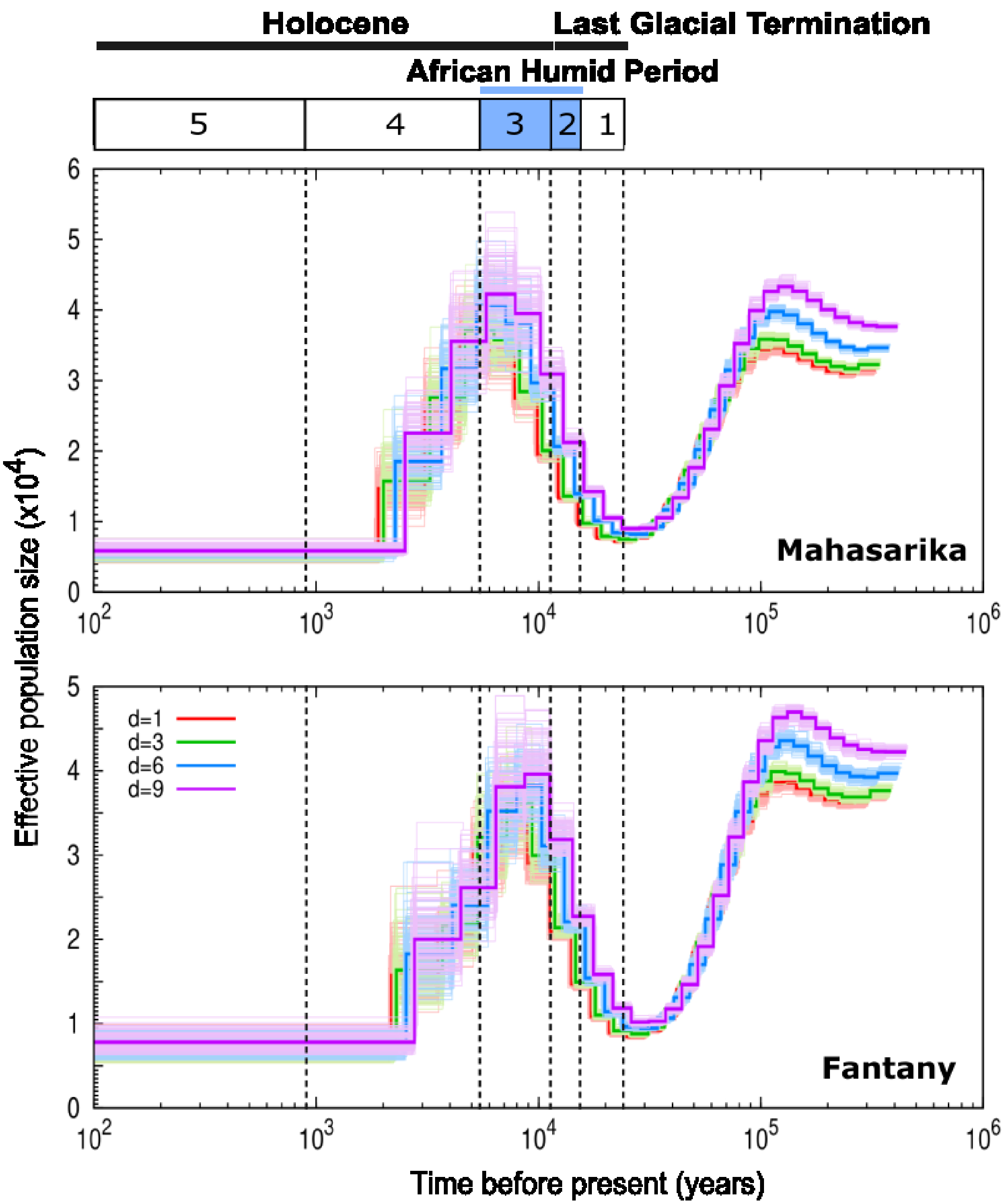

Fig. S18. **Impact of varying the minimum read depth (–d) in the *PSMC* analyses for Mahasarika (upper panel) and Fantany (lower panel)**. The demographic dynamics of *M. arnholdi* using different minimum read depth options (–d1, –d3, –d6, –d9) resulted in similar demographic curves for both study sites. The legend on the top identifies the main periods of vegetation changes derived from the paleoecological analyses (see main text). All the runs were performed considered a generation time of 2.5 years.

2.2.6. Impact of protein-coding sites in the demographic reconstructions of *M. arnholdi.* We reran the demographic analyses without the protein-coding sites to exclude possible confounding effects of sites under selection. We firstly removed all protein-coding sites from our aligned and trimmed BAM files according to the information available for the *Microcebus murinus* reference genome (GenBank Assembly accession number: GCA_000165445.3)[32]. We then reran the *PSMC, Stairway plot* and the best *fastsimcoal2* demographic model (Model 7) using the same options as in the previous analyses considering all sites. None of the new results showed deviations from previous results generated using the full dataset and included in the main text (Figure 3). For all new analyses, the *M. arnholdi* dynamics fall within the same paleoenvironmental periods as discussed in the main text (Fig. S19 – S21). For instance, the *PSMC* without the protein-coding sites confirms that Mahasarika and Fantany exhibited an increase of population size during the AHP, and suffered a population decline during the Mid-Holocene (Fig. S19).

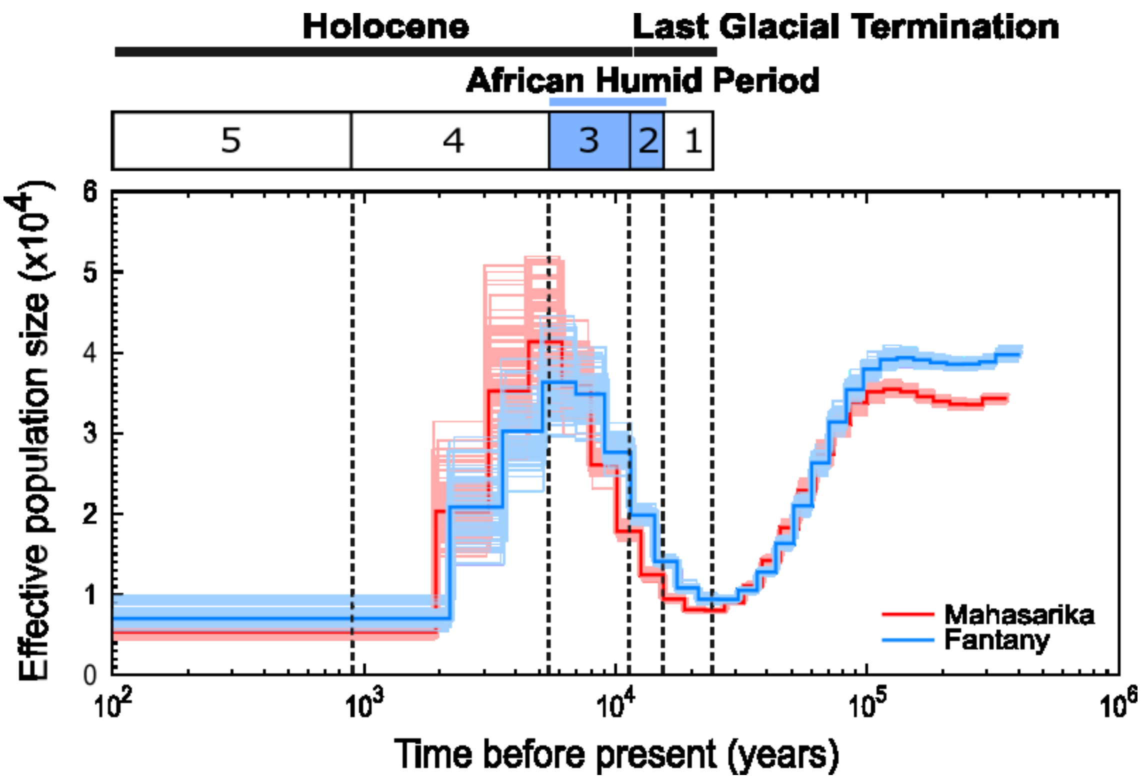

Fig. S19. Impact of the exclusion of protein-coding sites in the demographic history of *M. arnholdi* using *PSMC*. The thick red and blue lines represent the inferred trajectories of Mahasarika (red) and Fantany (blue) after the exclusion of the protein-coding sites. The results shows no deviation from the data generated using the full dataset (Figure 3a).

Knowing that sites violating Hardy–Weinberg equilibrium (HWE) often represent genotyping errors or sites under selection, we also repeated the *Stairway Plot* analyses without

sites deviating from HWE. We excluded sites displaying heterozygote excess with P-values significant at the 0.001 level using ANGSD[59]. The comparison of the *Stairway Plot* dynamics considering: (i) all sites, (ii) only sites in HWE, and (iii) non-protein coding sites resulted in identical demographic trends for the larger population (Fantany; N = 26; Fig. S20b). The results for the smaller population (Mahasarika; N = 12; Fig. S20a) were also similar, except for the old population dynamics (~ 100 kyr) when considering the dataset without protein-coding sites. This is likely an effect of the substantial reduction of the number of informative polymorphic sites (see Table S8 for details). To validate this hypothesis, we repeated the *Stairway Plot* analyses for Fantany considering both the full dataset and the dataset without protein-coding sites, and the same number of individuals than in Mahasarika (N = 12). The comparison of the four datasets (Fig. S20c) confirms that the reconstruction of the ancient dynamics at ~ 100 kyr is sensitive to both a small sample size and a reduced number of sites included in the analyses.

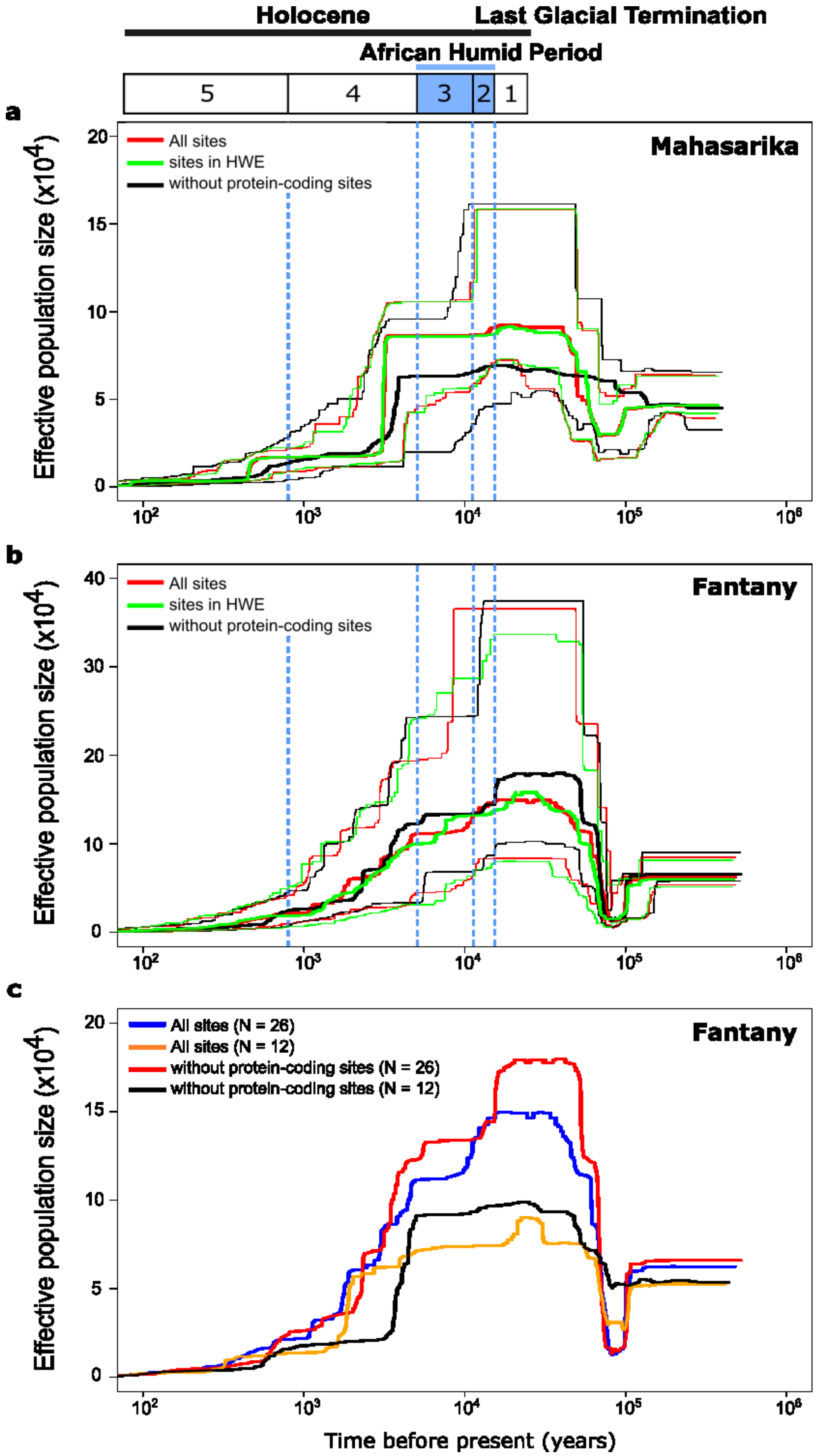

Holocene
Last Glacial Termination
African Humid Period
5
4
3
2
1
a
All sites
sites in HWE
without protein-coding sites
Mahasarika
Effective population size (x10^4)
b
All sites
sites in HWE
without protein-coding sites
Fantany
Effective population size (x10^4)
c
All sites (N = 26)
All sites (N = 12)
without protein-coding sites (N = 26)
without protein-coding sites (N = 12)
Fantany
Effective population size (x10^4)
Time before present (years)

Fig. S20. Impact of the exclusion of protein-coding sites in the demographic history of *M. arnholdi* inferred by the *Stairway Plot*. a, Impact of excluding protein-coding sites (black) and sites with deviation from Hardy-Weinberg Equilibrium (HWE, green) on the inferred trajectories of Mahasarika (N = 12). **b,** Impact of excluding protein-coding sites (black) and sites with deviation from HWE (green) on the inferred trajectories of Fantany (N = 26). **c,** Impact of sample size (N = 12 vs. N = 26) and the dataset (all sites vs. no protein-coding sites) on the demographic inferences of Fantany. The thick lines in Figures **a** and **b** correspond to the median effective population size and the light lines represent the 2.5 and 97.5% confidence intervals. The results show no deviation from the data generated with the full dataset for recent times, but suggest that larger datasets are essential to correctly infer older demographic dynamics.

The best *fastsimcoal2* demographic model (Model 7) rerun without the protein-coding sites suggests that the first population decline occurred ~ 5.4 kyr (i.e., at the termination of the AHP), and the second decline ~ 0.3 kyr (i.e., during the last millennium; Fig. S21a). These values approximates the previous time estimates using all sites (see Table S9), and are in concordance with our original interpretation that the first decline was likely triggered by the aridity increase, and the second decline was probably shaped by both climatic and anthropogenic impacts. Finally, we evaluated the fit of the expected pairwise 2d-SFS obtained for Model 7 and the observed 2d-SFS after removing the protein-coding sites. The results confirm the existence of a good fit (Fig. S21b). Altogether, we conclude that the demographic reconstructions of *M. arnholdi* were not impacted by sites violating the neutrality assumption.

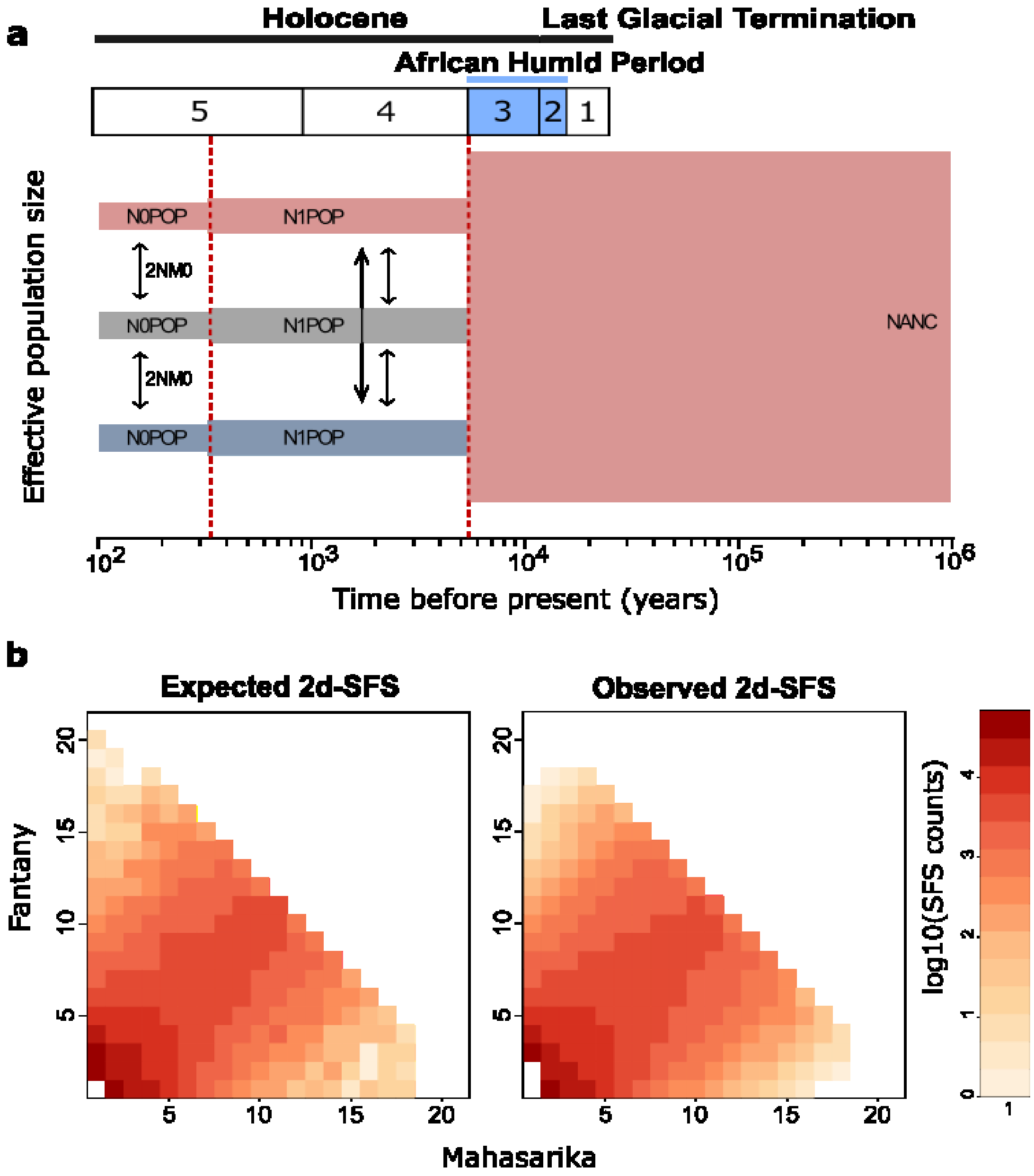

Fig. S21. Impact of the exclusion of protein-coding sites in the demographic modelling of *M. arnholdi* with *fastsimcoal2*. a, Illustration of the best demographic model (M7) after removal the protein-coding sites. The results confirms that the first population decline took place at the termination of the African Humid Period (~ 5.4 kyr; period 4) and the second decline occurred during the last millennium (~ 0.3 kyr; period 5). The different populations are represented by distinct colors. An additional population ("ghost population") was included to represent *M. arnholdi* sampling sites that were not covered by our sampling scheme. The width of bars is not exactly proportional to the estimated effective population (see Table S9 for details about the parameters estimation). The occurrence of gene flow is exemplified by arrows. N0POP = effective population size for each population at present time; N1POP = effective population size for each population after the first

population decline; NANC = ancestral population size; 2NM0 = average number of haploid immigrants entering the population per generation. T1 = time of the younger population decline; T2 = time of the older population decline (see Figure 3d). **b,** comparison of the expected pairwise 2d-SFS obtained for Model 7 and the observed 2d-SFS after the exclusion of the protein-coding sites. Each row shows the observed or expected 2d-SFS in log10 scale. Monomorphic sites across all samples were discarded. The expected SFS was multiplied by the total number of SNPs to be in the same scale as the observed 2d-SFS. The figure confirms that there is good fit between the Model 7 and the genomic data after the removal of the protein-coding sites (see Fig. S12).

Table S1. List of Accelerator Mass Spectrometry radiocarbon dating obtained from cores LM1A and LM1B. Measurements indicated in grey correspond to outliers.

Sample code	Core	Composite depth (cm)	Material	14C Age	Calibrated age (median)	Calibrated age (2σ)	C Mass
Poz-101233	LM1B	234.35	bulk	505±30	510	491-540	>1mgC
Poz-101234	LM1B	321.46	bulk	2225±30	2219	2099-2312	>1mgC
Poz-97327	LM1B	348.48	bulk	2685±30	2761	2731-2843	>1mgC
Poz-101235	LM1B	426.28	bulk	3775±35	4081	3935-4231	>1mgC
Poz-97328	LM1B	515.05	bulk	5230±40	5945	5761-6173	>1mgC
Poz-101236	LM1B	596.93	bulk	7800±40	8530	8428-8603	>1mgC
Poz-106018	LM1B	636.93	bulk	8830±50	9812	9565-10146	>1mgC
Poz-101237	LM1B	672.7	bulk	10230±50	11864	11630-12040	>1mgC
Poz-94285	LM1A	686.26	bulk	10300±60	11979	11753-12388	>1mgC
Poz-97329	LM1B	750.99	bulk	10280±60	11935	11650-12369	>1mgC
Poz-111303	LM1B	786.49	bulk	12120±60	13923	13764-14096	>1mgC
Poz-97330	LM1B	821.76	bulk	12260±60	14114	13865-14396	0.7mgC
Poz-111270	LM1B	869.1	bulk	12900±60	15339	15139-15599	>1mgC
Poz-106099	LM1B	905.24	bulk	12180±60	14005	13790-14168	0.65mgC
Poz-97331	LM1B	972.93	bulk	12930±60	15388	15170-15641	0.5mgC
Poz-111271	LM1A	998.39	bulk	31700±700	35652	34331-37444	0.3mgC
Poz-106100	LM1B	998.98	bulk	23310±300	27506	26996-27980	0.12mgC
Poz-106101	LM1B	1018.98	bulk	20430±250	24560	23970-25231	0.10mgC
Poz-101239	LM1B	1033.98	bulk	19820±120	23808	23490-24121	>1mgC

Table S2. List of individuals used for the three different demographic methods (*PSMC*, *Stairway Plot* and *fastsimcoal2*) and respective sampling locations.

Site	Sample ID	Latitude (°N)	Longitude (°E)	Sex	PSMC	Stairway Plot	*fastsimcoal2*
Mahasarika	M172	-12.522500	49.171503	M		X	X
Mahasarika	M176	-12.521762	49.171449	M		X	X
Mahasarika	M177	-12.522002	49.171540	M		X	
Mahasarika	F179	-12.513236	49.185247	F		X	X
Mahasarika	F182	-12.512337	49.187275	F		X	X
Mahasarika	F187	-12.512342	49.187331	F		X	
Mahasarika	F189	-12.512724	49.189859	F		X	X
Mahasarika	M197	-12.516145	49.177648	M		X	X
Mahasarika	M202	-12.515409	49.177699	M		X	X
Mahasarika	F208	-12.534563	49.172867	F	X	X	X
Mahasarika	M211	-12.530392	49.172056	M		X	X
Mahasarika	M217	-12.526261	49.175285	M		X	X
Fantany	F219	-12.691328	49.187968	F		X	
Fantany	F220	-12.690911	49.177168	F		X	
Fantany	F221	-12.691590	49.175873	F		X	X
Fantany	F222	-12.690769	49.176769	F		X	
Fantany	F225	-12.688768	49.173328	F		X	X
Fantany	F226	-12.690204	49.176118	F		X	
Fantany	M227	-12.690649	49.188085	M		X	
Fantany	M229	-12.692943	49.190754	M		X	
Fantany	F230	-12.692921	49.190785	F		X	X
Fantany	M232	-12.692398	49.190254	M		X	
Fantany	F233	-12.690946	49.188093	F		X	
Fantany	F234	-12.690867	49.188106	F		X	X
Fantany	M236	-12.690643	49.188069	M		X	
Fantany	F239	-12.692905	49.172407	F	X	X	
Fantany	M242	-12.692905	49.172407	M		X	
Fantany	F243	-12.692905	49.172407	F		X	X
Fantany	M245	-12.693038	49.172673	M		X	X
Fantany	M246	-12.691669	49.187035	M		X	X
Fantany	M247	-12.692562	49.190501	M		X	X
Fantany	M249	-12.692562	49.190501	M		X	
Fantany	M250	-12.689802	49.181150	M		X	X
Fantany	F252	-12.688783	49.178068	F		X	X
Fantany	F257	-12.687819	49.177150	F		X	
Fantany	M259	-12.687819	49.177150	M		X	
Fantany	M260	-12.687757	49.177510	M		X	

Fantany	M261	-12.687757	49.177510	M		X

Table S3. Information regarding the number of raw reads obtained from the RADseq sequencing: Illumina sequencing (# raw reads), number of reads that pass the quality filters (# reads after filtering), number of reads that were maintained after read alignment against the reference genome (# reads after filtering), and final number of reads that were kept for the downstream analyses (# reads without PCR duplicates) for the 38 *M. arnholdi* samples that passed all quality filters and were used for the demographic analyses.

Sample ID	# raw reads	# reads after filtering	# reads after mapping	# reads without PCR duplicates
M172	7195942	6432086	6045690	4340317
M176	7044038	6316228	5957032	4295324
M177	7486094	5973370	5649536	4506556
F179	6654996	5966592	5626661	4095313
F182	9493922	8487094	7894754	5571903
F187	8560786	7642090	7204389	5106322
F189	6145224	5497036	5208736	3806023
M197	5771052	5128518	4816559	3639983
M202	8094150	7239754	6882020	4940597
F208	5803452	5156734	4858065	3681512
M211	6403694	5073910	4750649	3787391
M217	5702156	5073106	4775024	3614356
F219	6727788	5840144	5477270	4527456
F220	7248420	6282072	5908053	4880074
F221	13463414	11746434	11127530	8543693
F222	15766560	13725764	14034	1572
F225	10844260	9409078	8816604	7112992
F226	14379062	12346268	11622481	9234538
M227	13814756	11945104	11328200	9012897
M229	9564618	8437460	6394881	4973663
F230	16428192	14341020	11575764	8761533
M232	6806066	5953496	4981055	4131346
F233	10400374	9016736	8507218	6889778
F234	17650798	15411746	11935391	8924663
M236	17388904	15174836	11308136	8365073
F239	10771680	9280946	8689703	7009404
M242	11373338	9028016	8456841	6869436
F243	10753442	9340896	8770279	7103831
M245	13177134	11382216	10758872	8605551
M246	10566840	9223788	8690425	7052052
M247	11031930	9573280	9009215	7283676
M249	13388930	11556332	10887707	8697992
M250	8392816	7286396	6842855	5628443

F252	9944552	8648434	8131199	6624522
F257	9746702	8440134	7948727	6463275
M259	9958622	8679180	8157264	6623857
M260	9001046	7837700	7366993	6029796
M261	7382390	6417050	6051393	4993984

Table S4. List of all demographic parameters used in each model during the *fastsimcoal2* analyses, and their respective search ranges. N0POP = effective population size for each population at present time; N1POP = effective population size for each population before a given demographic event; NANC = ancestral population size; NMAH, NFAU and NGHO = effective population size of populations Mahasarika, Fantany and Ghost, respectively; N1FAU = effective population size of Fantany after the first bottleneck (asymmetric model; M8). All population size parameters are given in haploid numbers. 2Nm = average number of haploid immigrants entering the population per generation, where 2NM0 denotes the present migration rate between demes and 2NM1 the ancient migration rate. T = time of a given demographic event, where T1 corresponds to the most recent event and T3 to the oldest one. Time estimates are given in number of generations.

				Search Range		
**Parameter**	**Models**	**Value**	**Distribution**	**Min.**	**Max.**	**Bounded?**
N0POP	1	Integer	Uniform	50	1 e5	no
N0POP	3, 4, 5, 6, 9, 10, 13	Integer	Uniform	15	1 e5	no
N0POP	2	Integer	Uniform	50	3 e5	no
N0POP	11	Integer	Uniform	15	1 e4	no
N0POP	7, 12	Integer	Uniform	10	1 e4	no
N1POP	7, 11, 12	Integer	Uniform	15	1 e5	no
N1POP	9	Integer	Uniform	50	3 e5	no
NANC	2, 6, 7, 8	Integer	Uniform	50	3 e5	no
NANC	9, 13	Integer	Uniform	15	1 e5	no
NANC	10, 12	Integer	Uniform	50	4 e5	no
2NM0	1, 2	Float	Log-Uniform	50	100	yes
2NM0	3, 4, 5, 6, 7, 8, 9, 10, 11, 12, 13	Float	Log-Uniform	0.001	20	yes
2NM1	5, 10, 11, 12, 13	Float	Log-Uniform	0.001	20	yes
T1	2, 3, 5, 6, 11	Integer	Uniform	100	5 e4	no
T1	7, 8, 9	Integer	Uniform	100	1000	no
T1	13	Integer	Uniform	100	6000	no
T1	10, 12	Integer	Uniform	100	1 e5	no
T2	7, 9	Integer	Uniform	1000	5 e4	no
T2	13	Integer	Uniform	6000	3 e5	no
T2	8	Integer	Uniform	1000	6000	no
T2	10, 12	Integer	Uniform	1 e5	5 e4	no
T3	8	Integer	Uniform	6000	5 e4	no
NMAH, NFAU, NGHO	8	Integer	Uniform	10	1 e4	no
N1FAU	8	Integer	Uniform	15	1 e5	no

Table S5. Demographic parameter estimates that maximized the likelihood of each of the 13 alternative demographic models after 100 independent simulations per model. N0POP = effective population size for each population at present time; N1POP = effective population size for each population before a given demographic event; NANC = ancestral population size. All population size parameters are given in haploid numbers. 2NM0 = average number of haploid immigrants entering the population per generation at present and 2NM1 = average number of haploid immigrants entering the population per generation before a given demographic event. T = time of a given demographic event, where T1 corresponds to the most recent event and T3 to the oldest one. Time estimates are given in number of generations. MIG = Migration rates scaled according to effective population sizes.

Category	Model	Topology	N0POP	N1POP	NANC	T1	T2	T3	2NM0	2NM1	MIG0	MIG1
Simple	M1	Null model	70 814	_	_	_	_	_	52.0	_	_	_
	M2	One size change	208 969	_	39 211	106	_	_	51.4	_	2.5E-04	_
	M3	Recently Structured	47 918	_	143 754	1 812	_	_	0.2	_	3.7E-06	_
	M4	Ancient structured	47 033	_	_	_	_	_	4.1	_	8.7E-05	_
	M5	Change in connectivity	37 230	_	_	217 383	_	_	4.2	0.05	1.14E-04	1.26E-06
Recently structured	M6	Recently structured + one bottleneck	7 800	_	159 874	737	_	_	3.8	_	4.88E-04	_
	M7	Recently structured + two bottlenecks	13 937	14 329	151 573	429	1 957	_	7.2	_	5.19E-04	5.05E-04
	M8	Recently structured + asymetric bottleneck	2 959 (MAH); 4 015 (FAU); 86 (GHO)	15 901 (N1FAU)	98 581	216	18 288	310 408	13.5	_	3.37E-03	8.50E-04
	M9	Old expansion + one bottleneck	6 862	148 284	160 215	560	25 212		3.4	_	4.94E-04	_
Change in connectivity	M11	Change + one bottleneck	21 416	160 930	_	5 611	_	_	4.2	1.2	1.96E-04	7.40E-06
	M10	One bottleneck + change	37 766	_	181 486	984	52 825	_	0.05	12.3	1.44E-06	3.27E-04
	M12	Two bottlenecks + change	19 928	214 419	255 633	5 227	148 692	_	4.1	3.3	2.07E-04	1.53E-05
	M13	Old expansion + change	39 300	_	27 715	9 611	426 988	_	4.2	0.2	1.06E-04	6.35E-06

Table S6. Rank of the 13 demographic models tested with *fastsimcoal2* based on the Akaike Information Criteria (AIC). Likelihoods were computed based on the parameters that maximized the likelihood of each model in a total of 100 independent simulations per model, in log10 units. Delta Likelihood (ΔLhood) represents the difference between the observed Likelihood and the maximum expected Likelihood based on 100 simulations. Delta AIC corresponds to the difference in AIC to the best model in each category. The three best ranked demographic models are highlighted by an asterisk.

Category	Model	Topology	Log10(Lhood)	Δlhood	# parameters	AIC	ΔAIC/category	Rank
Simple	M1	Null model	-3248795.7	29363.1	2	14963956.9	118382.0	13°
	M2	One size change	-3243516.2	24083.6	4	14939643.6	94068.7	12°
	M3	Recently structured	-3225580.8	6148.2	3	14857031.4	11456.5	10°
	M4	Ancient structured	-3225693.4	6260.8	2	14857547.8	11972.9	11°
	M5	Change in connectivity	-3223093.1	3660.5	4	14845574.9	0	8°
Recently structured	M6*	Recently structured + one bottleneck	-3222115.9	2683.3	4	14841073.9	833.9	3°
	M7*	Recently structured + two bottlenecks	-3222094.3	2661.7	6	14840978.5	738.5	2°
	M8*	Recently structured + asymmetric bottleneck	-3221932.7	2510.1	9	14840239.9	0	1°
	M9	Old expansion + one bottleneck	-3222153.9	2721.2	6	14841252.7	1012.7	4°
Change in connectivity	M10	One bottleneck + change	-3222832.0	3399.4	6	14844376.2	2967.4	7°
	M11	Change + one bottleneck	-3222188.2	2755.6	5	14841408.8	0	5°
	M12	Two bottlenecks + change	-3222201.3	2768.7	7	14841473.1	64.3	6°
	M13	Old expansion + change	-3223313.9	3881.2	6	14846595.7	5186.9	9°

Table S7. Maximum likelihood (ML) estimates for the demographic models M6 and M8, and respective 95% confidence intervals (CI) generated by block-bootstrap. All population size parameters are given in number of haploid copies. Time changes in years were scaled considering GT = 2.5 years. N0POP = effective population size for each population at present time; NANC = ancestral population size; NMAH, NFAU and NGHO = effective population size of populations Mahasarika, Fantany and Ghost, respectively; N1FAU = effective population size of Fantany after the first bottleneck (asymmetric model); 2NM0 = average number of haploid immigrants entering the population per generation; T = time of a given demographic event (in years), where T1 corresponds to the most recent event and T3 to the oldest event.

M6			
		95 % CI	
Parameter	ML estimate	Lower bound	Upper bound
N0POP	7 800	8 454	10 478
NANC	159 874	158 887	159 499
T1	1 843	2 178	2 778
2NM0	3.81	3.85	3.96

M8			
		95 % CI	
Parameter	ML estimate	Lower bound	Upper bound
N0MAH	2 959	5 111	7 307
NFAU	4 015	5 698	8 085
NGHO	86	526	813.0
NFAU	15 901	18 508	43 813
NANC	98 581	297 503	325 414
T1	540	3 440	5 693
T2	45 720	18 428	29 698
T3	776 020	1 122 485	1 133 605
2NM0	13.52	14.76	15.13

Table S8. Number of sites retained in our dataset for each demographic method when considering all sites and excluding protein-coding sites. The implementation of the HWE filter resulted in the removal of > 1% and ~ 5% of the polymorphic sites from the original Mahasarika and Fantany datasets, respectively. After excluding protein-coding sites, ~ 61% of the polymorphic sites were retained for Mahasarika and 58% for Fantany.

Study site	Dataset	# Total sites	# Polymorphic sites
Mahasarika (n = 12)	all sites	49,576,419	304,133
	sites in HWE	49,576,419	304,130
	without protein-coding sites	28,120,580	184,855
Fantany (n = 26)	all sites	58,219,228	490,778
	sites in HWE	56,848,952	468,453
	without protein-coding sites	32,224,550	284,334
Fantany (n = 12)	all sites	56,536,865	391,589
	without protein-coding sites	32,062,623	238,575

Table S9. Demographic parameters inferred under the best *fastsimcoal2* demographic model (Model 7) after exclusion of protein-coding sites and considering all sites. Maximum-likelihood (ML) estimates were obtained from the run with the highest composite likelihood. All population size estimates are given in number of haploid copies. Time changes were scaled considering GT = 2.5 years. N0POP = effective population size for each population after the most recent population decline; N1POP = effective population size for each population after the older population decline; NANC = ancestral population size; 2NM0 = average number of haploid immigrants entering the population per generation. T1 = time of the most recent population decline; T2 = time of the older population decline.

Parameter	ML estimate (without protein coding-sites)	ML estimate (all sites)
N0POP	6,839	13,937
N1POP	8,030	14,329
NANC	73,676	151,573
T1	322.5	1,073
T2	5,410	4,893
2NM0	3.7	7.23

SI References

1. Woodward, C. A. & Gadd, P. S. The potential power and pitfalls of using the X-ray fluorescence molybdenum incoherent: coherent scattering ratio as a proxy for sediment organic content. *Quat. Int.* **514**, 30–43 (2019).
2. Liu, X., Colman, S. M., Brown, E. T., Minor, E. C. & Li, H. Estimation of carbonate, total organic carbon, and biogenic silica content by FTIR and XRF techniques in lacustrine sediments. *J. Paleolimnol.* **50**, 387–398 (2013).
3. Ségalen, P. Etude des sols de la station des Quinquinas (montagne d'Ambre). *Mémoires l'Institut Sci. Madagascar. Série D Sci. la Terre* **3**, 165–179 (1951).
4. Simonneau, A. *et al.* Tracking Holocene glacial and high-altitude alpine environments fluctuations from minerogenic and organic markers in proglacial lake sediments (Lake Blanc Huez, Western French Alps). *Quat. Sci. Rev.* **89**, 27–43 (2014).
5. Rosenmeier, M. F. *et al.* Influence of vegetation change on watershed hydrology: implications for paleoclimatic interpretation of lacustrine δ18O records. *J. Paleolimnol.* **27**, 117–131 (2002).
6. Almquist-Jacobson, H., Almendinger, J. E. & Hobbie, S. Influence of terrestrial vegetation on sediment-forming processes in kettle lakes of west-central Minnesota. *Quat. Res.* **38**, 103–116 (1992).
7. Kasper, T. *et al.* Indian Ocean Summer Monsoon (IOSM)-dynamics within the past 4 ka recorded in the sediments of Lake Nam Co, central Tibetan Plateau (China). *Quat. Sci. Rev.* **39**, 73–85 (2012).
8. Hogg, A. G. *et al.* SHCal13 Southern Hemisphere calibration, 0-50,000 years cal BP. *Radiocarbon* **55**, 1889–1903 (2013).
9. Blaauw, M. Methods and code for 'classical'age-modelling of radiocarbon sequences. *Quat. Geochronol.* **5**, 512–518 (2010).
10. Davison, W. Iron and manganese in lakes. *Earth-Science Rev.* **34**, 119–163 (1993).
11. Dray, S., Dufour, A.-B. & others. The ade4 package: implementing the duality diagram for ecologists. *J. Stat. Softw.* **22**, 1–20 (2007).
12. Gosling, W. D., Miller, C. S. & Livingstone, D. A. Atlas of the tropical West African pollen flora. *Rev. Palaeobot. Palynol.* **199**, 1–135 (2013).
13. Rasoloarijao, T. M. *et al.* Pollen morphology of melliferous plants for Apis mellifera unicolor in the tropical rainforest of Ranomafana National Park, Madagascar. *Palynology* **43**, 292–320 (2019).
14. Schüler, L. & Hemp, A. Atlas of pollen and spores and their parent taxa of Mt Kilimanjaro and tropical East Africa. *Quat. Int.* **425**, 301–386 (2016).
15. Straka, H. & Friedrich, B. *Palynologia Madagassica et Mascarenica*. (Ed. du Muséum; Akademie der Wissenschaften und der Literatur; F. Steiner, 1988).
16. Guillaumet, J.-L., Betsch, J.-M. & Callmander, M. W. Renaud Paulian et le programme du CNRS sur les hautes montagnes à Madagascar: étage vs domaine. *Zoosystema* **30**, 723 (2008).
17. Du Puy, D. J. & Moat, J. Vegetation mapping and classification in Madagascar (using GIS): implications and recommendations for the conservation of biodiversity. *Chorology, Taxon. Ecol. floras Africa Madagascar* 97–117 (1998).
18. Trigui, S. M. *Etude floristique et biogéographique des altitudes supérieures de la Montagne d'Ambre (Nord de Madagascar)*. (Conservatoire et Jardin botaniques de la Ville de Genève, 2010).
19. Koechlin, J., Guillaumet, J.-L. & Morat, P. *Flore et végétation de Madagascar*. (1974).
20. Sayer, C., Roberts, N., Sadler, J., David, C. & Wade, P. M. Biodiversity changes in a shallow lake ecosystem: a multi-proxy palaeolimnological analysis. *J. Biogeogr.* **26**, 97–114 (1999).
21. Burney, D. A. Modern pollen spectra from Madagascar. *Palaeogeogr. Palaeoclimatol. Palaeoecol.* **66**, 63–75 (1988).
22. Burney, D. A., Robinson, G. S. & Burney, L. P. Sporormiella and the late holocene extinctions in Madagascar. *Proc. Natl. Acad. Sci. U. S. A.* **100**, 10800–10805 (2003).
23. Railsback, L. B. *et al.* Relationships between climate change , human environmental impact

, and megafaunal extinction inferred from a 4000-year multi-proxy record from a stalagmite from northwestern Madagascar. *Quat. Sci. Rev.* **234**, 106244 (2020).
24. Dewar, R. E. *et al.* Stone tools and foraging in northern Madagascar challenge Holocene extinction models. *PNAS* **110**, 12583–12588 (2013).
25. Grimm, E. C. CONISS: a FORTRAN 77 program for stratigraphically constrained cluster analysis by the method of incremental sum of squares. *Comput. Geosci.* **13**, 13–35 (1987).
26. Juggins, S. rioja: Analysis of Quaternary science data. (2015).
27. Rakotondravony, R. & Radespiel, U. T. E. Varying Patterns of Coexistence of Two Mouse Lemur Species (*Microcebus ravelobensis* and *M. murinus*) in a Heterogeneous Landscape. *Am. J. Primatoll* **938**, 928–938 (2009).
28. Seutin, G., White, B. N. & Boag, P. T. Preservation of avian blood and tissue samples for DNA analyses. *Can. J. Zool.* **69**, 82–90 (1991).
29. Davey, J. L. & Blaxter, M. W. RADseq: Next-generation population genetics. *Brief. Funct. Genomics* **9**, 416–423 (2010).
30. Bolger, A. M., Lohse, M. & Usadel, B. Trimmomatic: A flexible trimmer for Illumina sequence data. *Bioinformatics* **30**, 2114–2120 (2014).
31. Larsen, P. A. *et al.* Hybrid de novo genome assembly and centromere characterization of the gray mouse lemur (*Microcebus murinus*). *BMC Biol.* **15**, 1–17 (2017).
32. Lecompte, E., Crouau-Roy, B., Aujard, F., Holota, H. & Murienne, J. Complete mitochondrial genome of the gray mouse lemur, *Microcebus murinus* (Primates, Cheirogaleidae). *Mitochondrial DNA Part A* **27**, 3514–3516 (2016).
33. Li, H. *et al.* The sequence alignment/map format and SAMtools. *Bioinformatics* **25**, 2078–2079 (2009).
34. Metzker, M. L. Sequencing technologies — the next generation. *Nat. Rev. Genet.* **11**, 31 (2010).
35. Skotte, L., Korneliussen, T. S. & Albrechtsen, A. Estimating Individual Admixture Proportions from Next Generation Sequencing Data. **195**, 693–702 (2013).
36. Korneliussen, T. S., Albrechtsen, A. & Nielsen, R. ANGSD: Analysis of Next Generation Sequencing Data. *BMC Bioinformatics* **15**, 1–13 (2014).
37. Schmieder, R. & Edwards, R. Quality control and preprocessing of metagenomic datasets. *Bioinformatics* **27**, 863–864 (2011).
38. Li, H. & Durbin, R. Fast and accurate short read alignment with Burrows–Wheeler transform. *Bioinformatics* **25**, 1754–1760 (2009).
39. Blouin, M. S. DNA-based methods for pedigree reconstruction and kinship analysis in natural populations. *Trends Ecol. Evol.* **18**, 503–511 (2003).
40. Korneliussen, T. S. & Moltke, I. Sequence analysis NgsRelate : a software tool for estimating pairwise relatedness from next-generation sequencing data. *Bioinformatics* **31**, 4009–4011 (2015).
41. Liu, X. & Fu, Y.-X. Exploring Population Size Changes Using SNP Frequency Spectra. *Nat Genet.* **47**, 555–559 (2015).
42. Foote, A. D. *et al.* Genome-culture coevolution promotes rapid divergence of killer whale ecotypes. *Nat. Commun.* **7**, (2016).
43. Li, H. & Durbin, R. Inference of human population history from individual whole-genome sequences. *Nature* **475**, 493–496 (2011).
44. Meyer, A. L. S., Pie, M. R. & Passos, F. C. Assessing the exposure of lion tamarins (Leontopithecus spp.) to future climate change. *Am. J. Primatol.* **76**, 551–562 (2014).
45. Beichman, A. C., Phung, T. N. & Lohmueller, K. E. Comparison of Single Genome and Allele Frequency Data Reveals Discordant Demographic Histories. *G3 Genes, genomes, Genet.* **7**, 3605–3620 (2017).
46. Nadachowska-Brzyska, K., Burri, R., Smeds, L. & Ellegren, H. PSMC analysis of effective population sizes in molecular ecology and its application to black-and-white Ficedula flycatchers. *Mol. Ecol.* **25**, 1058–1072 (2016).
47. Salmona, J., Heller, R., Lascoux, M. & Shafer, A. Inferring demographic history using genomic data. in *Population Genomics* 511–537 (Springer, 2017).
48. Mazet, O., Rodríguez, W., Grusea, S., Boitard, S. & Chikhi, L. On the importance of being structured: Instantaneous coalescence rates and human evolution-lessons for ancestral

population size inference? *Heredity (Edinb).* **116**, 362–371 (2016).
49. Orozco-Terwengel, P. The devil is in the details: The effect of population structure on demographic inference. *Heredity (Edinb).* **116**, 349–350 (2016).
50. Excoffier, L., Dupanloup, I., Huerta-Sánchez, E., Sousa, V. C. & Foll, M. Robust Demographic Inference from Genomic and SNP Data. *PLoS Genet.* **9**, (2013).
51. Barratt, C. D. *et al.* Vanishing refuge ? Testing the forest refuge hypothesis in coastal East Africa using genome - wide sequence data for seven amphibians. *Mol. Ecol.* 4289–4308 (2018). doi:10.1111/mec.14862
52. Excoffier, L. & Foll, M. fastsimcoal : a continuous-time coalescent simulator of genomic diversity under arbitrarily complex evolutionary scenarios. *Bioinformatics* **27**, 1332–1334 (2011).
53. Chikhi, L. *et al.* The IICR (inverse instantaneous coalescence rate) as a summary of genomic diversity: Insights into demographic inference and model choice. *Heredity (Edinb).* **120**, 13–24 (2018).
54. Soraggi, S., Wiuf, C. & Albrechtsen, A. Powerful Inference with the D-Statistic on Low-Coverage Whole-Genome Data. *G3 Genes, genomes, Genet.* **8**, 551–566 (2017).
55. Fredsted, T., Pertoldi, C., Schierup, M. H. & Kappeler, P. M. Microsatellite analyses reveal fine-scale genetic structure in grey mouse lemurs (*Microcebus murinus*). *Mol. Ecol.* **14**, 2363–2372 (2005).
56. Radespiel, U., Jurić, M. & Zimmermann, E. Sociogenetic structures, dispersal and the risk of inbreeding in a small nocturnal lemur, the golden-brown mouse lemur (*Microcebus ravelobensis*). *Behaviour* **146**, 607–628 (2009).
57. Radespiel, U., Schulte, J., Burke, R. J. & Lehman, S. M. Molecular edge effects in the Endangered golden-brown mouse lemur *Microcebus ravelobensis*. *Oryx* **53**, 716–726 (2019).
58. Evanno, G., Regnaut, S. & Goudet, J. Detecting the number of clusters of individuals using the software STRUCTURE: A simulation study. *Mol. Ecol.* **14**, 2611–2620 (2005).
59. Meier, J. I. *et al.* Demographic modelling with whole-genome data reveals parallel origin of similar *Pundamilia* cichlid species after hybridization. *Mol. Ecol.* 123–141 (2017). doi:10.1111/mec.13838
