# Supplementary material for: Past environmental changes affected lemur population dynamics prior to human impact in Madagascar
Source: Commun Biol. 2021 Sep 15;4:1084. doi: 10.1038/s42003-021-02620-1 (PMC8443640; doi:10.1038/s42003-021-02620-1)
Supplement: Supplementary file 2 — Description of Supplementary Files [file 42003_2021_2620_MOESM2_ESM.pdf]

Description of Additional Supplementary Files

Filename: Supplementary Data 1
Description: Raw data for pollen analyses.

Filename: Supplementary Data 2
Description: Raw data for charcoal analyses.

Filename: Supplementary Data 3
Description: Raw data for grain size analyses.

Filename: Supplementary Data 4
Description: Raw data for XRF analyses.
