# Supplementary material for: Past environmental changes affected lemur population dynamics prior to human impact in Madagascar
Source: Commun Biol. 2021 Sep 15;4:1084. doi: 10.1038/s42003-021-02620-1 (PMC8443640; doi:10.1038/s42003-021-02620-1)
Supplement: Supplementary file 7 — Reporting Summary [file 42003_2021_2620_MOESM7_ESM.pdf]

nature research

Corresponding author(s): Ute Radespiel and Helena Teixeira

Last updated by author(s): Jul 16, 2021

Reporting Summary

Nature Research wishes to improve the reproducibility of the work that we publish. This form provides structure for consistency and transparency in reporting. For further information on Nature Research policies, see our Editorial Policies and the Editorial Policy Checklist.

Statistics

For all statistical analyses, confirm that the following items are present in the figure legend, table legend, main text, or Methods section.

n/a	Confirmed	
☐	☒	The exact sample size (n) for each experimental group/condition, given as a discrete number and unit of measurement
☐	☒	A statement on whether measurements were taken from distinct samples or whether the same sample was measured repeatedly
☒	☐	The statistical test(s) used AND whether they are one- or two-sided<br>*Only common tests should be described solely by name; describe more complex techniques in the Methods section.*
☒	☐	A description of all covariates tested
☐	☒	A description of any assumptions or corrections, such as tests of normality and adjustment for multiple comparisons
☐	☒	A full description of the statistical parameters including central tendency (e.g. means) or other basic estimates (e.g. regression coefficient) AND variation (e.g. standard deviation) or associated estimates of uncertainty (e.g. confidence intervals)
☒	☐	For null hypothesis testing, the test statistic (e.g. F, t, r) with confidence intervals, effect sizes, degrees of freedom and P value noted<br>*Give P values as exact values whenever suitable.*
☐	☒	For Bayesian analysis, information on the choice of priors and Markov chain Monte Carlo settings
☒	☐	For hierarchical and complex designs, identification of the appropriate level for tests and full reporting of outcomes
☒	☐	Estimates of effect sizes (e.g. Cohen's d, Pearson's r), indicating how they were calculated

Our web collection on statistics for biologists contains articles on many of the points above.

Software and code

Policy information about availability of computer code

Data collection	Not applicable.
Data analysis	The softwares used are described in detail in the Materials and methods section

For manuscripts utilizing custom algorithms or software that are central to the research but not yet described in published literature, software must be made available to editors and reviewers. We strongly encourage code deposition in a community repository (e.g. GitHub). See the Nature Research guidelines for submitting code & software for further information.

Data

Policy information about availability of data

All manuscripts must include a data availability statement. This statement should provide the following information, where applicable:

- Accession codes, unique identifiers, or web links for publicly available datasets
- A list of figures that have associated raw data
- A description of any restrictions on data availability

All RADseq sequences obtained in this study are publicly available at Sequence Read Archive (NCBI) in the BioProject PRJNA560399 (Number accession: SAMN14854044 – SAMN14854081). Whole Genome sequences are available in the BioProject PRJNA632451 (Biosample: SAMN14909740 for Mahasarika and SAMN14909741 for Fantany). Scripts used for all analyses are available upon request. The paleoenvironmental data are provided as supplementary data (see supplementary data file 1 to 4) and are deposited on PANGAEA open access database.

Field-specific reporting

Please select the one below that is the best fit for your research. If you are not sure, read the appropriate sections before making your selection.

☐ Life sciences ☐ Behavioural & social sciences ☒ Ecological, evolutionary & environmental sciences

For a reference copy of the document with all sections, see nature.com/documents/nr-reporting-summary-flat.pdf

Ecological, evolutionary & environmental sciences study design

All studies must disclose on these points even when the disclosure is negative.

Study description	We integrated high-resolution paleoenvironmental reconstructions derived from a 25,000 years sedimentary record in northern Madagascar with demographic inferences of an endemic forest-dwelling primate species (Microcebus arnholdi) to investigate how past environmental changes shaped current biodiversity in a tropical rainforest ecosystem.
Research sample	Microcebus arnholdi is an endemic primate species to Madagascar that is known to occur in the evergreen humid forest of Montagne d'Ambre in northern Madagascar. This species represents a suitable study model to investigate the impact of past environmental changes on demographic dynamics because (i) it is forest-dependent and should therefore be strongly influenced by vegetation changes; (ii) it has a comparably short generation time and a high reproductive rate, thereby quickly accumulating genetic signatures of past demographic events, and (iii) populations are still large enough and may have preserved enough genetic diversity to accurately reconstruct their demographic history. Furthermore, several natural lakes are occurring in Montagne d'Ambre and one them is occurring in the center of this National Park (Lake Maudit) which makes it an essential study site to obtain palaeoenvironmental reconstructions.
Sampling strategy	Two sites were selected for mouse lemur sample collection in Montagne d'Ambre National Park: an evergreen humid forest at the northern limit of the park (Mahasarika) and a forest/grassland ecotone at the southern limit (Fantany). In addition the volcanic maar lake located on the center of Montagne d'Ambre (L. Maudit) was selected for lacustrine coring.
Data collection	Mouse lemurs were sampled by Helena Teixeira and her field assistants. Small ear biopsies were taken from all captured animals for genomic analyses and stored in Queen's lysis buffer.<br>The lacustrine sediment core was sampled following standard methods by Vincent Montade, Laurent Bremond and Sandratrinirainy Ranarilalatiana.
Timing and spatial scale	Mouse lemurs were captured daily between August to October 2017 and the sediment core was sampled between May and June 2017.
Data exclusions	In order to control for genotyping errors, the genomic samples with a low mean sequencing coverage were removed from our dataset. Additionally, to overcome a potential demographic history reconstruction bias we estimated the relatedness among all the individuals in our dataset. Only one individual of each closely related dyad (e.g., parent-offspring or full siblings) was retained in our dataset. To obtain a correct age-depth model for palaeoenvironmental reconstructions, five radiocarbon dates, considered as outliers, were excluded from the age-depth model (see supplementary material for details).
Reproducibility	No manipulative experiments were carried out. We critically evaluated the impact of choosing different generation times in all performed demographic analyses (see section 2.2.4. of the supplementary material). In the demographic modelling with fastsimcoal2, a total of 100 independent runs were performed for each model to ensure convergence and high support for the results.
Randomization	Please see the Table S2 of the supplementary material for information about the number of individuals considered for each demographic method.
Blinding	Not applicable.

Did the study involve field work? ☒ Yes ☐ No

Field work, collection and transport

Field conditions	Field work was conducted during the dry season and temperature was fluctuating between 15 and 25°C.
Location	Field work for mouse lemur sampling took place in Mahasarika (-12.534 °N, 49.176 °E, 1,073 m asl) and Fantany (-12.696 °N, 49.167° E, 848 m asl). The lacustrine sediment core was sampled in Lac Maudit (-12.582°N, 49.150°E, 1,250 m asl).
Access & import/export	Data collection and exportation of both tissue samples and the sediment core were conduted with the permission of the following authorities or institutions: The Director of Montagne d'Ambre National Park, the Direction du Système des Aires Protégées, the Direction Générale du Ministère de l'Environnement et des Forêts de Madagascar, Madagascar's Ad Hoc Committee for Fauna and Flora, and the Organizational Committee for Environmental Research (research permits N°78/17/MEEF/SG/DGF/DSAP/SCB.Re and N° 79/17 MEEF/SG/DGF/DSAP/SCB/RE). Export permission was granted by the authorities in Madagascar under the permit number 855C-EA10-MG17. Import permission was granted by the Bundesamt für Naturschutz, Germany, under the permit number DE-E-06530/17.

Disturbance	Not applicable.

Reporting for specific materials, systems and methods

We require information from authors about some types of materials, experimental systems and methods used in many studies. Here, indicate whether each material, system or method listed is relevant to your study. If you are not sure if a list item applies to your research, read the appropriate section before selecting a response.

Materials & experimental systems

n/a	Involved in the study
☒	☐ Antibodies
☒	☐ Eukaryotic cell lines
☒	☐ Palaeontology and archaeology
☐	☒ Animals and other organisms
☒	☐ Human research participants
☒	☐ Clinical data
☒	☐ Dual use research of concern

Methods

n/a	Involved in the study
☒	☐ ChIP-seq
☒	☐ Flow cytometry
☒	☐ MRI-based neuroimaging

Animals and other organisms

Policy information about studies involving animals; ARRIVE guidelines recommended for reporting animal research

Laboratory animals	Not applicable.
Wild animals	A total of 46 Microcebus arnholdi were captured by hand near the trees that they used during nighttime activity. The animals were kept in a Sherman Trap with a piece of banana and moved to the camping site where they were handled during the next morning. All the animals were released at dusk on the day of handling at the same place where they were captured. Small ear biopsies were taken from all captured animals for genomic analyses.
Field-collected samples	Tissue samples were stored in Queen's lysis buffer and preserved at room temperature during the field season and were frozen at -20°C afterwards until extraction.
Ethics oversight	This study was conducted in agreement with the laws of Madagascar and adhered to the principles of the Code of Best Practices for Field Primatology of the International Primatological Society and the ethical guidelines of the Association for the Study of Animal Behaviour and the Animal Behaviour Society. All capture and handling procedures followed routine protocols and were approved by the Malagasy Authorities and by the Institute of Zoology, University of Veterinary Medicine Hannover.

Note that full information on the approval of the study protocol must also be provided in the manuscript.
